# Mono-n-hexyl phthalate: exposure estimation and assessment of health risks based on levels found in human urine samples

**DOI:** 10.1007/s00204-024-03835-x

**Published:** 2024-08-17

**Authors:** Ralph Pirow, Ulrike Bernauer, Annegret Blume, Adrian Cieszynski, Gabriele Flingelli, Astrid Heiland, Matthias Herzler, Bettina Huhse, Christian Riebeling, Esther Rosenthal, Moustapha Sy, Thomas Tietz, Achim Trubiroha, Andreas Luch

**Affiliations:** German Federal Institute for Risk Assessment (BfR), Max-Dohrn-Strasse 8-10, 10589 Berlin, Germany

**Keywords:** Di-n-hexyl phthalate, Mono-n-hexyl phthalate, Tolerable daily intake, Human biomonitoring data, Benchmark dose modelling, Reverse dosimetry

## Abstract

Mono-n-hexyl phthalate (MnHexP) is a primary metabolite of di-n-hexyl phthalate (DnHexP) and other mixed side-chain phthalates that was recently detected in urine samples from adults and children in Germany. DnHexP is classified as toxic for reproduction category 1B in Annex VI of Regulation (EC) 1272/2008 and listed in Annex XIV of the European chemical legislation REACH; thereby, its use requires an authorisation. Health-based guidance values for DnHexP are lacking and a full-scale risk assessment has not been carried out under REACH. The detection of MnHexP in urine samples raises questions about the sources of exposure and concerns of consumer safety. Here, we propose the calculation of a provisional oral tolerable daily intake value (TDI) of 63 µg/kg body weight/day for DnHexP and compare it to intake levels corresponding to levels of MnHexP found in urine. The resulting mean intake levels correspond to less than 0.2% of the TDI, and maximum levels to less than 5%. The TDI was derived by means of an approximate probabilistic analysis using the credible interval from benchmark dose modelling of published ex vivo data on reduced foetal testosterone production in rats. Thus, for the dose associated to a 20% reduction in testosterone production, a lower and upper credible interval of 14.9 and 30.0 mg/kg bw/day, respectively, was used. This is considered a conservative approach, since apical developmental endpoints (e.g. changed anogenital distance) were only observed at higher doses. In addition, we modelled various scenarios of the exposure to the precursor substance DnHexP from different consumer products, taking measured contamination levels into account, and estimated systemic exposure doses. Of the modelled scenarios including the application of sunscreen (as a lotion or pump spray), the use of lip balm, and the wearing of plastic sandals, and considering conservative assumptions, the use of DnHexP-contaminated sunscreen was highlighted as a major contributing factor. A hypothetical calculation using conservative assumptions for the latter resulted in a margin of safety in relation to the lower credible interval of 3267 and 1007 for adults and young children, respectively. Most importantly, it was found that only a fraction of the TDI is reached in all studied exposure scenarios. Thus, with regard to the reported DnHexP exposure, a health risk can be considered very unlikely.

**Supplementary Information:**

The online version contains supplementary material available at 10.1007/s00204-024-03835-x.

## Introduction

Esters of phthalic acid (phthalates) are mainly used as plasticisers in plastics such as PVC. They are not covalently bound in the respective plastics matrix and, therefore, can be released from it. Since phthalates are produced and used in large quantities, they can be detected almost everywhere in the environment (Net et al. 2015). REACH Annex XIV currently compiles 15 phthalates as toxic for reproduction and thus requiring authorisation. Only for bis(2-ethylhexyl) phthalate (EC no. 204-211-0, DEHP) and dibutyl phthalate (EC no. 201-557-4, DBP) authorisations have been granted for limited industrial applications. Moreover, the entries 51 and 52 of REACH Annex XVII prohibit the use of several phthalates in toys, childcare articles, and other consumer articles. Furthermore, the Regulation (EC) No 1223/2009 on cosmetic products lists 15 phthalates in Annex II, banning their use in cosmetic products. In the area of food safety, the EU Regulation (EU) No 10/2011 permits the use of a small number of phthalates for limited applications.

The detection of mono-n-hexyl phthalate (EC no. 246-302-8, MnHexP) has recently been reported in follow-up examinations of urine samples from children by the Federal State Office of Nature, Environment and Consumer Protection of North Rhine-Westphalia (LANUV 2024). The substance was also detected in urine samples from adults (UBA 2024a) as part of the ongoing sixth German Environmental Survey (GerES VI) conducted by the German Federal Environment Agency (UBA) (UBA 2024b). MnHexP could occur as primary metabolite from exposure to di-n-hexyl phthalate (EC no. 201-559-5, DnHexP) and certain other mixed-chain phthalates (BfR 2024). Moreover, DnHexP and mixed side-chain phthalates were detected in urine samples from seven-year-old children from Denmark in 2019 (Vilmand et al. 2023).

Secondary metabolites such as 5-hydroxy hexyl phthalate (5HO-MnHexP) and 5-carboxy pentyl phthalate (5cx-MnPentP) can be formed from MnHexP through further metabolic steps. MnHexP, 5HO-MnHexP and 5cx-MnPentP were proposed as biomarkers for exposure to DnHexP as part of the European Human Biomonitoring Initiative (HBM4EU 2022). The proportion of children aged between 2 and 6 years with detectable MnHexP concentrations in urine increased from 26% (66 of 251 children) in the 2017/2018 cross-section to 61% (152 of 205 children) in the 2020/2021 cross-section (LANUV 2021, 2022, 2024). DnHexP has been identified as substance of very high concern by ECHA in 2013 (ECHA 2013) and is included in the Authorisation List (REACH Annex XIV) since 2020 (ECHA 2020). Of the phthalates permitted under EU Regulation (EU) No 10/2011, none features hexyl side chains.

Both MnHexP and DnHexP do not have active registration as substances in use in the EU under REACH. To date, officially defined limits or health-based guidance values have not been derived in the EU Chemicals legislations for MnHexP or the possible parent compound DnHexP.

As part of a risk assessment, we evaluated the available toxicological studies performed with DnHexP and derived a value for the provisional oral tolerable daily intake (TDI) for humans. This TDI is provisional because certain studies that would be required for, e.g. higher tonnage level substances under REACH are currently not available. DnHexP is one of the phthalates that possess alkyl side chains in the critical C4–C6 alkyl backbone length range (Fabjan et al. 2006). Members of this group of phthalates with structural and metabolic similarities all show a comparable spectrum of effects in male rats, referred to as “phthalate syndrome” (ECHA 2016), albeit with different individual potencies.

The MnHexP content detected in the urine samples was also reassessed on the premise that the “phthalate syndrome” applies to it, too. For this purpose, a reverse dosimetry approach was applied to estimate the daily intake of DnHexP from the measured MnHexP concentrations using toxicokinetic assumptions in a back-calculation of concentrations in urine to the hypothetically corresponding external oral intake.

The cause of the presence of MnHexP in urine samples of adults and children is unknown. Possible sources such as DnHexP-contaminated sunscreen and other consumer products are being discussed. DnHexP itself is banned as an ingredient in cosmetic products, but might be introduced as an impurity. One such possible source is a specific, approved UV filter used, e.g. in sunscreen (CVUA Karlsruhe 2024). Moreover, an enquiry by the German Federal Office of Consumer Protection and Food Safety (BVL) to the responsible state authorities revealed occasional presence in consumer products. The highest amount of DnHexP has been found in plastic sandals.

Here, we have modelled four scenarios of exposure: dermal application of sunscreen, oral exposure after application of lip balm, as well as using a pump spray on the face, adding inhalation exposure. In addition, exposure has been modelled for wearing DnHexP-contaminated plastic sandals.

## Materials and methods

### Reconstruction of exposure to DnHexP using MnHexP levels detected in urine

The exposure to DnHexP was reconstructed from human biomonitoring (HBM) data provided by LANUV, which determines the exposure of children in day-care centres to harmful substances, including phthalates, at regular 3-year intervals. MnHexP was found in a follow-up analysis of urine samples from the cross-sectional studies conducted in 2017/18 and in 2020/21 (LANUV 2021, 2022, 2024). Following the approach used for other phthalates (Frederiksen et al. 2011; Koch et al. 2007), reverse dosimetry was applied to estimate the daily intake rate *DI* (µg/kg bw/day) of DnHexP from the concentration *C*_U_ (μg/L) of MnHexP in urine:

1 $$DI={C}_{\text{U}}\cdot \frac{{Q}_{\text{U}}}{{f}_{\text{UE}}}\cdot \frac{{MW}_{\text{p}}}{{MW}_{\text{m}}},$$

where *Q*_U_ (L/kg bw/day) is the average daily urinary output rate adjusted to body weight. *Q*_U_ values of 0.025 and 0.03 L/kg bw/day were assumed for adults and children, respectively (Table 1). *MW*_p_ and *MW*_m_ are the molecular weights of the parent substance (DnHexP) and the metabolite (MnHexP), respectively. The parameter *f*_UE_ is defined as the (molar) fraction of the oral dose of the parent substance that is excreted (within 24 h) via urine as metabolite (UBA 2011). For *f*_*UE*_  a value of 0.69 was used (see Supplemental Information), which was determined for the monoester metabolite of DBP (Anderson et al. 2001; Koch et al. 2007).

**Table 1:** Parameters for the calculation of the daily intake of DnHexP from the concentration of MnHexP in urine and vice versa

Parameter	Unit	Description	Value	Comment
*C*_U_	µg/L	Concentration of MnHexP in urine		See results
*Q*_U_	L/kg bw/day	Urinary output rate adjusted to bw	0.025	Adults (1.5 L/day, 60 kg bw)
			0.030	1–3-year-old children (Aylward et al. 2015; Lange et al. 2021)
*MW*_p_	g/mol	Molecular weight of DnHexP	334.45	
*MW*_m_	g/mol	Molecular weight of MnHexP	250.29	
*f*_UE_	-	Fraction of urinary excretion	0.69	See text

### Derivation of a provisional tolerable daily intake (TDI)

#### Dose–response assessment using benchmark dose modelling

Benchmark dose (BMD) modelling was applied to the data on the foetal testosterone production rate ex vivo from Saillenfait et al. (2013) to derive a point of departure (PoD) as the starting point for the risk assessment (see Supplemental Information for a more detailed justification of this PoD). To establish a benchmark response (BMR) for this endpoint, EFSA’s tiered approach for continuous data was followed (EFSA 2022). As biologically relevant BMR for the endpoint has not yet been established, a BMR was defined on the basis of the effect size theory for continuous data (Slob 2017). This theory requires the BMR to be defined in relation to the maximum response in an endpoint, which makes BMR values defined for endpoints with different maximum responses comparable, i.e. they can be regarded as equivalent in quantitative terms. The calculations were performed using the EFSA web-tool for Bayesian BMD Analysis of EFSA (2024) and Verlinden et al. (2024) using the default settings (except for the critical effect size, see “Results”). The R package BMABMDR version 0.0.0.9077 (Kremer et al. 2024) was used for the underlying calculations.

BMD modelling was applied to data on foetal testosterone production rate ex vivo, which were illustrated in Fig. 1 of Saillenfait et al. (2013) and were kindly provided by Dr. Sophie Ndaw of the French National Research and Safety Institute for the Prevention of Occupational Accidents and Diseases (INRS). Foetuses from 8 to 12 litters (three foetuses per litter; two hormone measurements per foetus from the left and right testes) were used to determine the testicular testosterone production. The litter was considered as statistical unit.

**Fig. 1:**
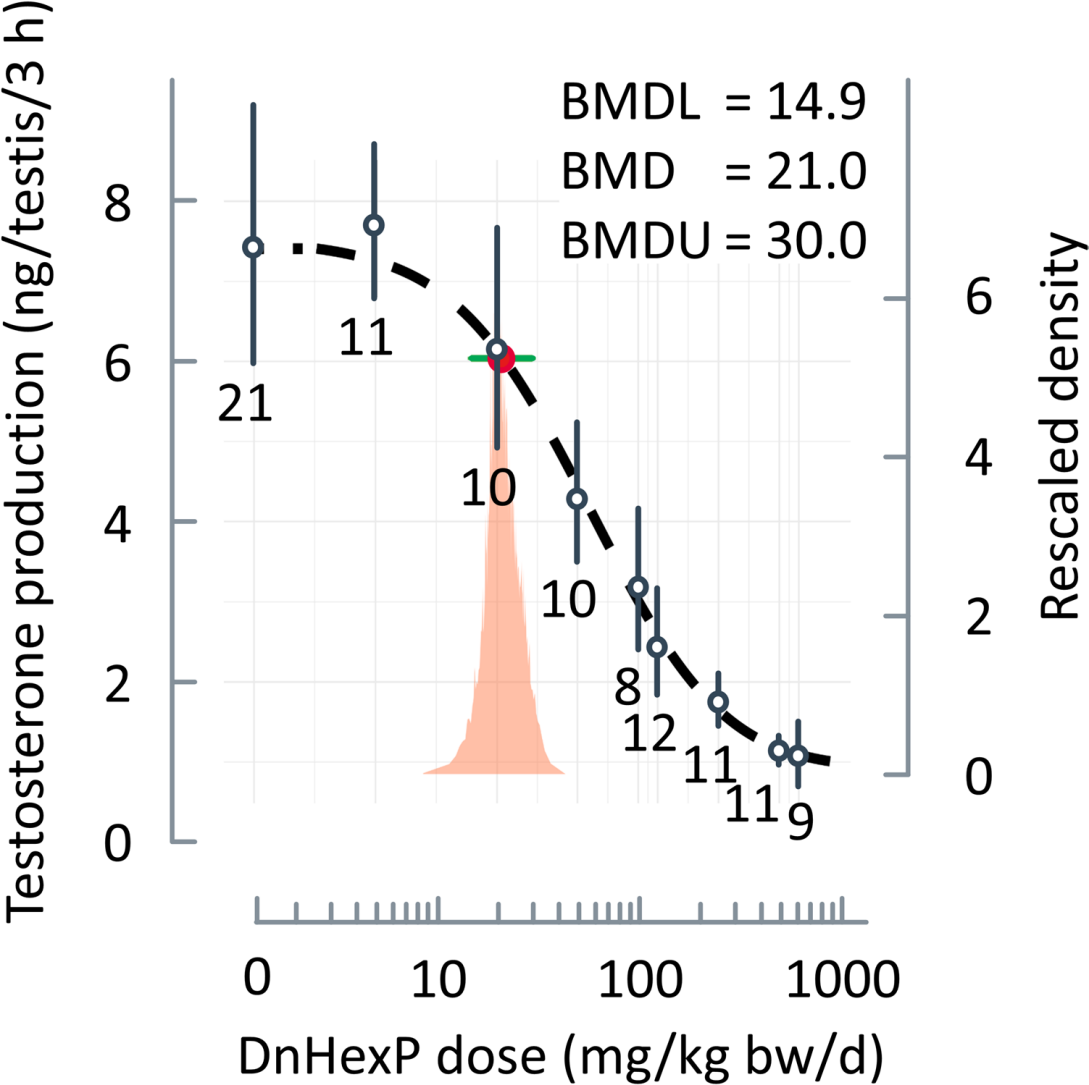
Dose–response curve modelled using Bayesian model averaging. Foetal testosterone production ex vivo is plotted as a function of the DnHexP dose administered by gavage to pregnant Sprague–Dawley rats (Saillenfait et al. 2013). Circles and error bars show the geometric means and the (arithmetic) standard deviations, respectively. Figures below the error bars indicate the number of analysed litters per group. The dashed curve shows the result of BMD modelling using Bayesian model averaging. The red dot and the green bar indicate the model-averaged BMD and the corresponding 95% credible interval, respectively, for a benchmark response (BMR) of 20%. Values for the BMD and for the lower and upper bounds (BMDL, BDMU) of credible interval are given. The orange area represents the density distribution around the BMD

The data are constituted of two experiments that are covering eight doses plus control, ranging from 0 to 625 mg/kg bw/day, with higher doses from 125 mg/kg bw/day originating exclusively from experiment 1. Foetal testosterone production in the controls was 7.73 ± 0.96 (mean ± standard deviation, *N* = 10 litters) in experiment 1 and 7.47 ± 2.08 (*N* = 11) in experiment 2. The difference between the two controls was not statistically significant (Welch two-sample *t*-test for unequal variances, *p* value = 0.72), which justifies to combine and analyse the data together.

In the present analysis, the data from both experiments were considered together (Table S1), which implies that experiment-specific variability was not accounted for. This choice is supported by preliminary analyses (Fig. S1), which revealed that the joint use of both experiments led to a more optimal behaviour of the dose–response curve (between 0 and 5 mg/kg/day and compared to experiment 1) and to reduced uncertainty (in comparison to experiments 1 and 2) on the estimated BMD and its 90% credible interval due to a better coverage of the range of tested doses.

#### Calculation of the TDI using approximate probabilistic analysis

An approximate probabilistic analysis was performed based on the corresponding guideline of WHO (2018) and has been described in detail elsewhere (BfR 2023).

### Assessment of the exposure to DnHexP from consumer products

To identify possible sources of exposure, data from the literature on the presence of DnHexP in consumer products and house dust as well as the concentrations of the substance in these media measured by the German surveillance authorities were collected. House dust can be ingested mostly by young children because of their mouthing behaviour, but it is also an identifier of the presence of such products in the households.

Based on the available data, four exposure scenarios were considered for assessing the exposure to DnHexP from consumer products: dermal application of sunscreen, oral exposure after application of lip balm, inhalation exposure after applying a pump spray for the face, and dermal exposure to plastic sandals.

For cosmetics, the exposure assessments followed the SCCS Notes of Guidance (NoG) (SCCS 2023), which were developed specifically for ingredients of cosmetic products. The NoG specify relevant exposure scenarios including parameter settings that need to cover “reasonably foreseeable conditions of use” in line with Article 3 of the Cosmetics Regulation (EC) No 1223/2009. For the dermal and oral exposure scenarios, the systemic exposure dose *SED* (µg/kg bw/day) was calculated by the following equation:

2 $$SED=\frac{{m}_{\text{prod}}\cdot c}{BW}\cdot {f}_{\text{abs}},$$

where *m*_prod_ (g) refers to the dermally applied (sunscreen) or unintentionally ingested (lip balm) product amount, *c* (mg/kg) is the concentration of DnHexP in the product, *f*_abs_ describes the absorption fraction for the dermal or oral route, and *BW* (kg) is the body weight. Parameter values are given in Table 2. A dermal absorption fraction of 0.05% was assumed for DnHexP. This figure is based on (i) the cumulative dermal absorption of 18% over 7 days following the application of ^14^C-labelled DnHexP under occlusive conditions in rats (Elsisi et al. 1989), and (ii) on the fact that rat skin is approximately four times more permeable than human skin (Pakalin et al. 2008; SCCP 2007). For sunscreen, the applied amount was set to 18 g/day for adults in line with the NoG (SCCS 2023). For young children (0–3 years), a value (on a per body weight basis) of 0.971 g/kg bw/day was assumed, which corresponds to the 95th percentile of the application rate for children in this age group (Ficheux et al. 2016).

**Table 2:** Parameters for the calculation of the systemic exposure dose from the exposure to DnHexP from consumer products

Parameter	Unit	Description	Value	Comment
*m*_prod_	g	Product amount	18 0.057 1.54	Dermal application of sunscreen Unintentional ingestion of lip balm Sprayed amount of product
*c*	mg/kg	Concentration of DnHexP	16	Maximum value (Fig. S2)
*f*_abs_	–	Absorption fraction	0.05 1	Dermal route Oral route (default)
*f*_air_	–	Airborne fraction	0.2	Default for a pump spray
*f*_ret_	–	Fraction of DnHexP retention in lung	0.75	Default for a pump spray
*r*_inh_	L/min	Inhalation rate	13	For adults
*V*_A_	L	Volume of Box A	1000	SCCS (2021)
*V*_B_	L	Volume of Box B	10,000	SCCS (2021)
*t*_A_	min	Duration of exposure in Box A	2	SCCS (2021)
*t*_B_	min	Duration of exposure in Box B	10	SCCS (2021)
*t*_contact_	h	Duration of dermal contact	10	
*A*_skin_	cm^2^	Exposed skin area	300	Soles of the feet of 10-year-olds
*M*_*R*_	µg/cm^2^/h	Migration rate	0.007	Figure 2
*BW*	kg	Body weight	60 10 22	Default for adults 3-year-old children 10-year-old children

The inhalation exposure via face pump spray was calculated following the three-step approach taken by the SCCS for the safety assessment of the UV filter homosalate (SCCS 2021). In a first step, the amount of DnHexP available for inhalation *a*_expo_ (µg) was calculated from the sprayed amount of product (*m*_prod_), the concentration (*c*) in the product, and the airborne fraction *f*_air_, which is the fraction of the non-volatile product that becomes airborne as droplets after spraying:

3 $${a}_{\text{expo}}={m}_{\text{prod}}\cdot c\cdot {f}_{\text{air}}.$$

Parameter values are given in Table 2. In a second step, the potential DnHexP amount inhaled was calculated by the 2-Box model (Steiling et al. 2014) that assumes two different zones (Box A and Box B) in which the emitted material is homogeneously distributed. Box A stands for the breathing zone accounting for the short term “near field” exposure, whereas Box B stands for the rest of the room (e.g. a smaller bathroom) and accounts for a longer “far field” scenario. Given the volumes *V*_A_ and *V*_B_ (L) for the two boxes, the duration of exposure *t*_A_ and *t*_B_ (min) in each box, and assuming no air exchange between the Box B and the environment, the potential DnHexP amount inhaled *a*_inh_ (µg) is calculated by

4 $${a}_{\text{inh}}={a}_{\text{expo}}\cdot {r}_{\text{inh}}\cdot \left(\frac{{t}_{\text{A}}}{{V}_{\text{A}}}+\frac{{t}_{\text{B}}}{{V}_{\text{B}}}\right),$$

where *r*_inh_ (L/min) is the inhalation rate (i.e. minute volume) of the exposed individual. Parameter values are given in Table 2. In a third step, the *SED* for the inhalation pathway is calculated by

5 $$SED=\frac{{a}_{\text{inh}}}{BW}\cdot {f}_{\text{ret}},$$

where *f*_ret_ is the fraction of DnHexP retention in the lung (i.e. inhaled minus exhaled). Only a small fraction of this *SED* initially retained in the lungs is respirable (depending on the size of the aerosol particles of the spray), while the largest, non-respirable fraction is coughed up or swallowed as a result of the lung’s clearance mechanism. As a conservative assumption, the non-respirable fraction was considered to be completely swallowed and thus systemically available.

**Fig. 2:**
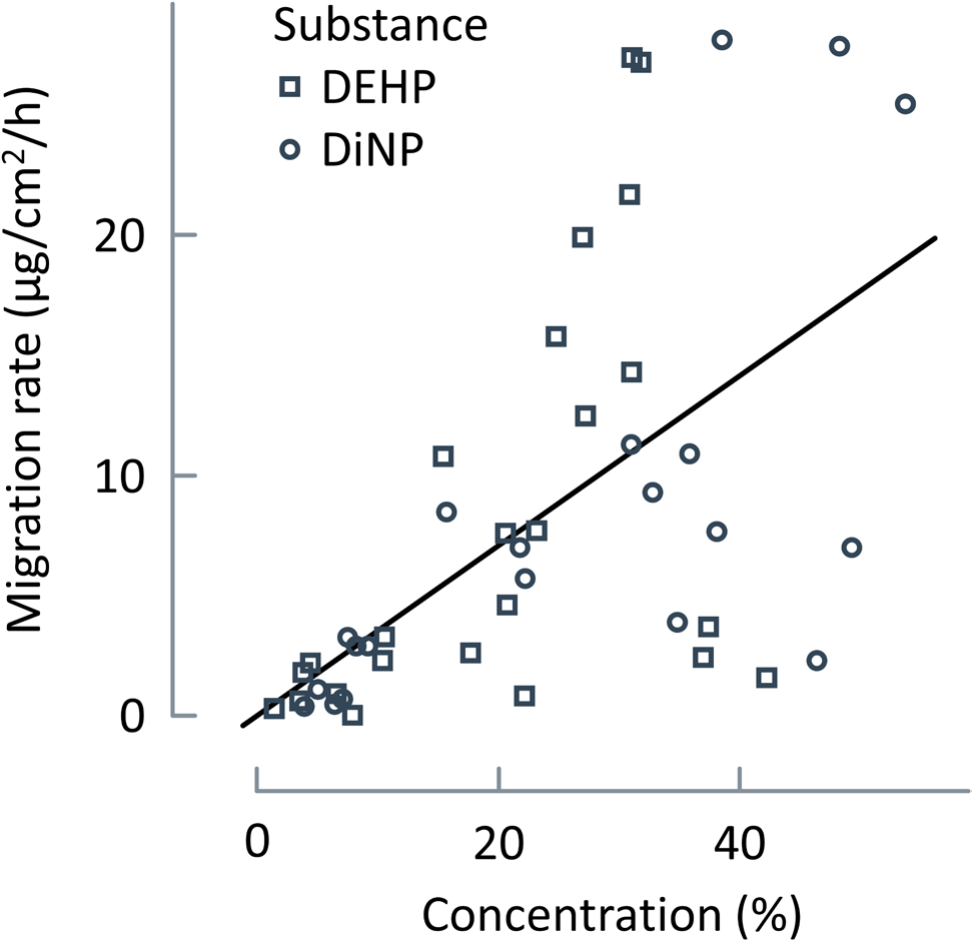
Correlation of the measured migration rates (µg/cm^2^/h) and concentrations of DEHP (circles) and DiNP (squares) from 138 diverse plastics consumer products (data are taken from Pfaff et al. (2012)). The migration was measured using sweat and saliva simulants. The solid line represents the result of the linear regression, yielding a value of *M*_R_ = 0.007 µg/cm^2^/h

For the dermal exposure to DnHexP released from plastic sandals, the SED was calculated as

6 $${SED}_{\text{dermal}}=\frac{{A}_{\text{skin}}\cdot {M}_{\text{R}}\cdot {t}_{\text{contact}}\cdot {f}_{\text{abs}}}{BW},$$

where *A*_skin_ (cm^2^) is the exposed skin surface area, *M*_R_ (μg/cm^2^/h) the migration rate at which DnHexP is released from the plastic material and transferred to the skin, and *t*_contact_ describes the duration of dermal contact. Parameter values are listed in Table 2. The *M*_R_, a value of 0.007 µg/cm^2^/h was estimated from a linear regression analysis of the data shown in Fig. 2.

### Conversion of systemic exposure doses to urine levels (using forward dosimetry)

A systemic exposure dose (*SED*) of DnHexP was converted into a concentration (*C*_U_) of MnHexP in urine by the following equation:

7 $${C}_{\text{U}}=SED\cdot \frac{{MW}_{\text{m}}}{{MW}_{\text{p}}}\cdot \frac{{f}_{\text{UE}}^{\text{SED}}}{{Q}_{\text{U}}},$$

where ${f}_{\text{UE}}^{SED}$ refers to the (molar) fraction of *SED* that is excreted via urine as MnHexP. The value for ${f}_{\text{UE}}^{SED}$ was assumed to be the same as that for *f*_UE_.

## Results

### Reconstruction of exposure to DnHexP using MnHexP levels detected in urine

Based on the concentration of MnHexP in morning urine samples of 2- to 6-year-old day-care children from the cross-sectional studies performed in 2017/2018 and 2020/2021 (LANUV 2024), the daily intake rates were estimated using Eq. 1 and the parameter values in Table 1. Median values were estimated at below 0.01 µg/kg bw/day (2017/2018 cross-section; median concentration below the limit of quantification) and 0.032 µg/kg bw/day (2020/2021 cross-section) (Table 3). The 95th percentile (P95) increased from 0.047 µg/kg bw/day (cross-section 2017/2018) to 0.507 µg/kg bw/day (cross-section 2020/2021).

**Table 3:** Estimated daily intake of DnHexP in µg/kg bw/day, calculated using volume-based urine concentrations of MnHexP from two cohorts of children (LANUV 2024)

Cohort	Size	50th percentile (median)	95th percentile	Maximum reported value
2017/2018	251	0.006	0.047	0.47
2020/2021	250	0.032	0.507	2.68

An interim analysis of data from the ongoing GerES VI study comprising 750 morning urine samples in adults revealed a maximum MnHexP concentration of 45.7 μg/L (HBM Commission 2024), which corresponded to a daily oral exposure of 2.21 µg/kg bw/day, which is lower than the maximum value for children from the 2020/2021 cross-section.

### Derivation of a provisional tolerable daily intake (TDI)

#### Selected study

The prenatal developmental toxicity study by Saillenfait et al. (2013) was selected as the basis for deriving a provisional TDI (see Supplemental Information). In their publication, the authors presented the results of two experiments in which Sprague–Dawley rats were administered 0, 5, 20, 50, and 100, and 0, 125, 250, 500, and 625 mg DnHexP/kg bw/day, respectively, by gavage on gestation days 12–19 and determined a no observed (adverse) effect level (NO(A)EL) of 5 mg/kg bw/day that was based on reduced foetal testosterone production ex vivo.

#### Benchmark dose modelling

For the endpoint “foetal testosterone production rate ex vivo”, the maximum response (*M*) consists of a five–tenfold decrease (Furr et al. 2014; Saillenfait et al. 2011); this corresponds to a reduction in the testosterone production rate to 10–20% compared to the control. According to the formula *ES* = *M*^1/8^ (Slob 2017), an equivalent effect size (*ES*) of small amplitude results in values of 1.223 and 1.334 for a fivefold and tenfold maximum change, respectively. The equivalent small amplitude effect size would, therefore, be a 1.223–1.334-fold decrease in testosterone production rate compared to the control, which corresponds to a reduction in testosterone production rate by 18–25%.

According to EFSA’s tiered approach, the biological relevance or degree of adversity must be taken into account for the (critical) effect size range derived in this way. For the endpoint “foetal testosterone production rate ex vivo”, by expert judgement a BMR of 20% was considered to be appropriate with regard to adverse effects and biological variance (see Supplemental Information).

Consequently, the dose–response relationship of the foetal testosterone production rate from Saillenfait et al. (2013) was subjected to BMD modelling with a BMR of 20% (Fig. 1). This yielded a BMD_20_ of 21.0 mg/kg bw/day and lower and upper limits of the credible interval around the BMD estimated of 14.9 (BMDL_20_) and 30.0 mg/kg bw/day (BMDU_20_), respectively. The credible interval includes the LO(A)EL of 20 mg/kg bw/day, whereas the NO(A)EL of 5 mg/kg bw/day is below the BMDL_20_. The BMDL_20_ of 14.9 mg/kg bw/day was then identified as the most relevant and robust PoD.

#### Calculation of the TDI

The provisional TDI for DnHexP was derived from the BMDL_20_ value using approximate probabilistic analysis and accounting for inter- and intra-species differences following the standard assessment factors proposed by the WHO (2018). A body weight of *BW* = 200 g was used for rats in accordance with the information in Saillenfait et al. (2013) and a body weight of *BW* = 60 kg was assumed for humans. Using the BMDL_20_ value, a 90% credible interval of 63–2780 µg DnHexP/kg bw/day was derived for the maximum safe daily intake. The lower limit of the credible interval of 63 µg/kg bw/day was then used as the TDI for the further risk assessment. The derived TDI is regarded provisional because of the limited data considered for the TDI derivation for DnHexP.

#### Comparison to the TDI value derived by EFSA for phthalates

In 2019, EFSA reassessed the health effects of five phthalates approved for use used in plastic food contact materials (DBP, benzyl butyl phthalate (EC no. 201-622-7, BBP), DEHP, diisononyl phthalate (EC no. 249-079-5, DiNP), diisodecyl phthalate (EC no. 247-977-1, DiDP)) (EFSA 2019). Four of these phthalates (DBP, BBP, DEHP and DiNP) were considered as a group in the assessment because they share the same mechanism of action and show a range of related reproductive toxicity effects. EFSA, therefore, established a group-TDI for this group of phthalates.

The different potencies of the four phthalates were taken into account by expressing the group-TDI in DEHP equivalents. For all four phthalates together, the TDI is 50 µg DEHP equivalents/kg bw/day. For a risk assessment, exposure data for each of the four phthalates must be converted to DEHP equivalents (EFSA 2019).

Subsequently, the total exposure in DEHP equivalents is compared with the TDI. The weighting factors are an expression of the differences in potencies, e.g. the weighting factor 5 for DBP indicates that this substance has five times the potency of DEHP.

DnHexP shares the same mechanism of action as DEHP. It is, therefore, justified to estimate the fraction of the group-TDI DnHexP accounts for. A weighting factor for DnHexP of 0.79 results from the ratio of the TDI for DEHP (50 µg/kg bw/day) and the preliminary TDI for DnHexP (63 µg/kg bw/day).

### Assessment of the exposure to DnHexP from consumer products

#### Exposure estimation for a hypothetical contamination of sunscreen with DnHexP

An exposure assessment was performed for the hypothetical case that consumers would use a sunscreen containing 10% of a UV filter which in turn would be contaminated with 0.3% DnHexP. The DnHexP concentration in such a sunscreen would consequently be 0.03%, i.e. 300 mg/kg. Using Eq. 2 and the parameter values in Table 2 for the dermal exposure scenario, the systemic exposure dose (*SED*) is estimated at 4.5 µg/kg bw/day for adults. For young children (0–3 years), an *SED* is estimated of 14.6 µg/kg bw/day. The *SED* values for this hypothetical contamination of sunscreen would correspond to the fraction of the provisional TDI of 7.1% (adults) and 23.3% (children).

#### Exposure from cosmetic products (sunscreen, lip balm, face pump spray)

A contamination with DnHexP of 16 mg/kg is assumed for the cosmetic products sunscreen, lip balm and pump spray, based on the maximum value reported for 57 samples of sunscreen products from 2020 to 2023 (CVUA Karlsruhe 2024) (Fig. S2). The dermal exposure of adults and young children to this DnHexP level in sunscreen resulted in an *SED* of 0.24 and 0.78 µg/kg bw/day, respectively.

For the oral exposure of adults following the application of lip balm, an ingested amount of 57 mg/day and an oral absorption fraction of 1 were assumed (Table 2). Applying Eq. 2 yields an *SED* of 0.015 µg/kg bw/day. For a 3-year-old child with a body weight of 10 kg, by assuming the same ingested amount as for adults, the SED is estimated at 0.091 µg/kg bw/day.

The inhalation exposure of adults via a pump spray for the face was estimated in a stepwise manner using Eqs. 3–6 and the parameter values in Table 2. By assuming that both the respirable and non-respirable fractions of the DnHexP initially retained in the lung become systemically available as a conservative approach, the *SED* is estimated at 0.0024 µg/kg bw/day. For a 3-year-old child with a body weight of 10 kg, by assuming the same inhaled amount as for adults, the SED is estimated at 0.014 µg/kg bw/day.

An aggregated *SED* of 0.2574 µg/kg bw/day is estimated for adults and 0.885 µg/kg bw/day for young children, respectively, by summing up the systemic exposure doses estimated from the different exposure pathways (dermal application of sunscreen, oral ingestion of lip balm, and inhalation of face pump spray). The aggregated *SED* values for this measured contamination of cosmetics would correspond to the fraction of the provisional TDI of 0.4% (adults) and 1.4% (children).

#### Dermal exposure to plastic sandals

Data on the presence of DnHexP in several consumer products were obtained following an enquiry by the German Federal Office of Consumer Protection and Food Safety (BVL) to the responsible state authorities. The samples came from Schleswig–Holstein/Bremen and were analysed within the frame of the North German cooperation by the state laboratory Berlin–Brandenburg as a follow-up to an investigation already conducted in 2021. DnHexP was found in plastic sandals, a skipping rope and bicycle handles in concentrations of 90–200 mg/kg, 110 mg/kg and 160 mg/kg, respectively. Using the highest content of 200 mg/kg in plastic sandals, an *SED* of 0.048 µg/kg bw/day was estimated using Eq. 6 and parameter values in Table 2 for a 6-year-old child with a body weight of 22 kg, wearing plastic sandals for 10 h.

### Conversion of systemic exposure doses to urine levels (using forward dosimetry)

#### From DnHexP in sunscreen and other cosmetic products to MnHexP levels in urine

Using Eq. 7 and the parameter values in Table 1, the *SED* values estimated for DnHexP in sunscreen were converted into MnHexP concentrations in urine. For the hypothetical contamination of sunscreen with 300 mg/kg DnHexP, the MnHexP concentration in urine was estimated to be 93 µg/L (adults) and 251 µg/L (1–3-year-old children). These hypothetical values are significantly higher than the maximum urine concentration value of 46.18 µg/L reported by LANUV (2024) and the maximum of 45.7 µg/L from the interim analysis of the GerES VI data (HBM Commission 2024).

For the measured maximum DnHexP contamination of 16 mg/kg in sunscreen, the MnHexP concentrations were 4.96 µg/L for adults and 13.4 µg/L for children (Table 4). The exposures to other cosmetic products (lip balm, face pump spray) with the same level of DnHexP contamination translated into MnHexP concentrations in urine being 1–2 orders of magnitude lower than those for sunscreen. The aggregated *SED* values would result in urine concentrations of 5.31 µg/L for adults and 18.3 µg/L for children, which are in the range of the values reported in LANUV (2024) or measured in the GerES VI study (HBM Commission 2024).

**Table 4:** Overview of the estimated systemic exposure and the derived concentrations of MnHexP in urine for adults and young children

Scenario	Exposure route	*SED* (µg/kg bw/day)	[MnHexP]_urine_ (µg/L)
Adults			
Sunscreen	Dermal	0.24	4.96
Lip balm	Oral	0.015	0.31
Pump spray for face	Inhalation	0.0024	0.05
Young children			
Sunscreen	Dermal	0.78	13.4
Lip balm	Oral	0.091	1.56
Pump spray for face	Inhalation	0.014	0.24
Plastic sandals	Dermal	0.048	1.48

#### From DnHexP in plastic sandals to MnHexP level in urine

The systemic exposure dose estimated at 0.048 µg/kg bw/day resulted in a MnHexP concentration in urine of 1.48 µg/L. In comparison, the level of MnHexP in the urine of 2–6-year-old children reported by LANUV was 0.56 µg/L (median) and 1.93 μg/L for the 75th percentile in the 2020/2021 cross-section (LANUV 2024).

## Discussion

### Risk characterisation based on dose reconstruction from MnHexP contents in urine

Based on the toxicological studies available for DnHexP, we calculated a provisional tolerable daily oral intake (TDI) of 63 µg DnHexP/kg bw/day. As the point of departure (PoD), we used a BMDL_20_ of 14.7 mg/kg bw/day based on decreased foetal testosterone production determined ex vivo by Saillenfait et al. (2013). An intake of DnHexP at the level of the TDI is currently considered not to present a health risk.

It should also be noted that the approximate probabilistic approach used here is more conservative than the standard “deterministic” approach to chemical risk assessment (dividing the PoD by a standard factor of 100). The deterministic approach would result in a provisional TDI of 147 µg/kg bw/day by dividing the BMDL_20_ by an overall assessment factor of 100 for inter- and intra-species variability.

Based on the urine levels of MnHexP determined in the cross-section 2021/2022 of the day-care centre study (LANUV 2024), we estimated the oral daily intake of DnHexP that would correspond to these urine contents. A comparison of the provisional TDI for DnHexP with the estimated DnHexP intake levels shows that the median, P95, and maximum intake levels correspond to 0.05%, 0.8%, and 4.3%, respectively, of the provisional TDI. Likewise, for the GerES VI study (HBM Commission 2024), the maximum DnHexP intake level was estimated to account for 3.5% of the provisional TDI. Moreover, in comparison with the EFSA group-TDI (EFSA 2019) of 50 μg DEHP equivalents/kg bw/day, the modelled DnHexP exposure represents only a small contribution. Based on dose reconstruction from measured MnHexP levels in urine, it can be concluded that DnHexP does not significantly contribute to the total intake of phthalates that are toxic to reproduction.

### Risk characterisation based on exposure modelling

Based on model calculations of systemic exposure doses, we determined that when using sunscreen containing up to 10% of UV filter contaminated with up to 0.3% DnHexP (i.e. a sunscreen contaminated with a maximum of 0.03% DnHexP), there is a sufficiently large margin of safety (MoS) in relation to the PoD, which amounts to 3267 and 1007 for adults and young children, respectively. Thus, a health risk with regard to DnHexP can be considered very unlikely.

Theoretically, the same UV filter could be used in several cosmetic products. For a cumulative exposure across all cosmetics except sunscreen, the SCCS assumes an application of 17.79 g/day. If this daily applied product amount is added to the 18 g/day assumed for sunscreen, the internal exposure would approximately be doubled. However, the MoS would still be around 1640 for adults and 503 for children.

Converting the systemic exposure doses resulting from the use of sunscreen contaminated hypothetically with 0.03% of DnHexP into MnHexP concentrations in urine yielded values of 93 µg/L (adults) and 251 µg/L (children), exceeding even the highest measured MnHexP values from the day-care centre study and GerES VI. However, the DnHexP contamination levels reported to date by the German surveillance authorities in samples of sunscreen are well below the assumed hypothetical DnHexP content in sunscreen (0.03%), showing a maximum of 0.0016% (Fig. S2). Systemic exposure doses based on this real contamination translated into urinary MnHexP concentrations of 4.96 µg/L (adults) and 13.4 µg/L (children), being ninefold and threefold lower, respectively, than the measured maximum levels. Therefore, the possibility of exposure by other sources has to be considered. Additional scenarios, including using lip balm, using a pump spray for the face, and wearing plastic sandals were modelled. The results demonstrate that urinary MnHexP levels predicted from these exposure scenarios can only explain a fraction of the measured values.

### Presence of DnHexP in house dust and consumer goods

We identified studies from other European and non-European countries in which the detection of DnHexP in house dust, children’s toys and children’s clothing is reported. In a literature search on the possible origin of MnHexP, only references relevant to DnHexP could be identified.

DnHexP was present in 90% of 30 PVC children’s toys tested in Turkey in 2020 (Akkbik et al. 2020). The EU limit of 0.1% (w/w) was exceeded in three cases (0.116, 0.144 and 0.185%). Measurements in children’s clothing from Asian countries in 2020 found DnHexP levels to be particularly high in cotton–spandex blends, although overall DnHexP was a minor phthalate (Tang et al. 2020).

Furthermore, in 60 households in Slovenia, DnHexP was detected in 95% of the samples up to 12.3 µg DnHexP/g house dust in 2015, suggesting that the substance could be present in products used in these households (Demirtepe et al. 2021). An initial investigation of the GerES VI (personnel communication) shows that the substance has also been measured in smaller quantities in German households. DnHexP was also detected in house dust in Asia, the USA and the Middle East (Al Qasmi et al. 2019; Ali et al. 2023; Basaran et al. 2020; Dodson et al. 2015; Guo and Kannan 2011; Kubwabo et al. 2013). Accumulation in house dust can be caused by, e.g. abrasion of products such as textiles and children’s toys. However, the exact source of the substance in house dust/urine could not be identified from these reports.

Irrespective of these assessments, DnHexP is classified as toxic for reproduction according to the CLP Regulation, and its presence in consumer products is undesirable. Further investigations could be carried out to determine additional sources of DnHexP and, if necessary, to eliminate them.

### Uncertainties in the assessment

The proposed PoD (decreased foetal testosterone production) for DnHexP is derived from a robust study and represents a well-established key event of male reproductive toxicity of C4–C6 phthalates. Nonetheless, there is some uncertainty with this PoD, since a NOAEL could not be determined in other studies, which were not taken forward for risk assessment because of shortcomings and reliability issues (see Supplemental Information). Furthermore, the data base for DnHexP is limited in comparison to other phthalates such as DEHP, in particular with regard to the window/duration of exposure (e.g. lack of multi-generation studies) and endpoints investigated.

Model parameters for dose reconstruction and for the conversion of systemic exposure doses into urinary MnHexP concentrations are subject to biological variability and uncertainty. Both may influence the results of the present analysis towards an underestimation or overestimation of the outcome variables. The urinary output rate *Q*_U_, which is the 24-h urine volume normalised to body weight, is highly dependent on individual characteristics. This parameter can, therefore, fluctuate because of both inter-individual variability (as body weight and urine production vary from person to person) and intra-individual variability, as urine production can vary greatly from day to day and from one person to another.

The fraction of urinary excretion *f*_UE_ and the related ${f}_{\text{UE}}^{SED}$ are crucial model parameters. The former is related to the oral dose of the parent substance (DnHexP), which is excreted (within 24 h) via urine as metabolite (MnHexP), while the latter is related to the systemic exposure dose. For an oral bioavailability of close to 100%, which applies to many phthalates, both parameters are equal. For *f*_UE_, we assumed a value of 0.69 that was experimentally determined for the monoester metabolite of DBP (Anderson et al. 2001), which shares with DnHexP the linear alkyl side chains. Taking for DnHexP the *f*_UE_ value from DBP is supported by toxicokinetic evidence on urine metabolite composition. Our analysis of the data of a toxicokinetic study in humans with oral administration of DBP (Koch et al. 2012) revealed that the monoester metabolite accounted for more than 70% of the total molar concentration of all analysed metabolites (see Supplemental Information). In the day-care centre study (LANUV 2024), the two secondary metabolites 5HO-MnHexP and 5cx-MnPentP were measured in addition to the primary metabolite MnHexP. Our analysis of the individual data from children in whom all three metabolites could be quantified showed that MnHexP was the main metabolite in urine samples with MnHexP concentrations greater than 2 μg/L. MnHexP accounted for more than 70% of the total molar concentration of the three metabolites. Unless other conceivable metabolites (5-oxo-mono-n-hexyl phthalate, monoesters with shortened β-oxidised side chains, phthalic acid) are formed in relevant quantities, and unless the excretion of the metabolites (including glucuronides) via the bile and subsequently via faeces does not make a relevant contribution compared to renal excretion, the *f*_*UE*_ and ${f}_{\text{UE}}^{SED}$ values for DnHexP can be expected be in the range of 0.69 or higher.

An additional uncertainty is that the metabolite spectrum in the urine may differ depending on the exposure route (oral, dermal, inhalation), so that different, exposure-related ${f}_{\text{UE}}^{SED}$ values are conceivable. Competing reactions of phase-I metabolism (ester cleavage as well as omega-, omega-1-, and β-oxidation of the side-chains), and absorption through hepatic and lymphatic routes following oral exposure could be responsible for the fact that the metabolite composition in urine after oral administration differs from that after dermal (and inhalation) exposure. Indeed, our analysis of reported urine metabolite compositions in toxicokinetic studies in humans with exposure to DEHP via different routes (Koch et al. 2004, 2005; Krais et al. 2018) revealed that the proportion of the monoester was threefold higher following a combined dermal and inhalation exposure in comparison to oral exposure (see Supplemental Information). In the case of dermal exposure, the ester cleavage of the phthalate appears to be the dominant first step with formation of the monoester, which can then be further glucuronidated, but can also be transported in the unconjugated form via albumin in blood (similar to the free fatty acids), before becoming excreted.

Phthalate metabolites have short elimination half-lives of 3–8 h in the human body (Anderson et al. 2011; Kessler et al. 2012; Koch et al. 2012). The back-calculation of metabolite levels in single spot or morning urine samples to the total amount of phthalate ingested during the day is, therefore, necessarily subject to uncertainties. This uncertainty is higher for oral exposures than for dermal exposures, for which the slow absorption from the skin rather than the serum elimination kinetics can be the rate-limiting step that governs the disappearance from the body. Moreover, the amount of phthalates taken up on different days can vary greatly. In addition taking into account the impact of fluid intake and sweating on urinary output rate, extreme values of metabolite concentrations in urine in particular should be interpreted with caution. As a consequence, particularly high metabolite levels in spot or morning urine samples generally lead to a significant overestimation of the 24-h intake as well as the long-term intake. Measures of central tendency such as the median, on the other hand, appear to be reasonably comparable (Aylward et al. 2017; Fisher et al. 2015; Frederiksen et al. 2013; Koch et al. 2017). Finally, uncertainties may occur when quantifying the MnHexP concentration *C*_U_ in urine. In the present analysis, however, it was assumed that these have a negligible influence on the estimates of the daily intake of DnHexP.

The assumed dermal absorption fraction *f*_abs_ of 0.05 is probably an overestimation. According to the study by Elsisi et al. (1989), less than 6% of the dermal dose of DnHexP was excreted in the urine of rats within the first 2 days after the start of dermal administration. This indicates that dermal absorption is slow. Given that rat skin is approximately four times more permeable than human skin (Pakalin et al. 2008; SCCP 2007), dermal absorption in humans is more likely to be approximately 1.5%.

Since phthalates with C4-C6 side-chains share a common mechanism of action, an additive effect can be assumed. The safety assessment described refers only to DnHexP in cosmetic products. Background contamination with other phthalates or from other sources was not taken into account. Relevant impurities must also be addressed in the safety assessment, which must be carried out by the producer for both ingredients and the product before a cosmetic product is made available on the market.

## Supplementary Information

Below is the link to the electronic supplementary material.

Supplementary file1 (DOCX 937 KB)
