# Supplementary material for: Mono-n-hexyl phthalate: exposure estimation and assessment of health risks based on levels found in human urine samples
Source: Arch Toxicol. 2024 Aug 17;98(11):3659–71. doi: 10.1007/s00204-024-03835-x (PMC11489165; doi:10.1007/s00204-024-03835-x)
Supplement: Supplementary file 1 — Supplementary file1 (DOCX 937 KB) [file 204_2024_3835_MOESM1_ESM.docx]

**Supplemental Information to**

**Mono-n-Hexyl phthalate: exposure estimation and assessment of health risks based on levels found in human urine samples**

Ralph Pirow* (ORCID 0000-0003-0513-1045), Ulrike Bernauer, Annegret Blume, Adrian Cieszynski, Gabriele Flingelli, Astrid Heiland, Matthias Herzler (ORCID 0000-0001-5636-8152), Bettina Huhse, Christian Riebeling (ORCID 0000-0003-1991-0170), Esther Rosenthal, Moustapha Sy (ORCID 0000-0001-9461-7316), Thomas Tietz (ORCID 0000-0002-7909-8420), Achim Trubiroha, Andreas Luch* (ORCID 0000-0002-5866-901X)

German Federal Institute for Risk Assessment (BfR),

Max-Dohrn-Strasse 8-10, 10589 Berlin, Germany

**Corresponding authors**

Ralph Pirow, PhD

Andreas Luch, MD, PhD

Bundesinstitut für Risikobewertung

Max-Dohrn-Str. 8-10

10589 Berlin

Germany

Tel. +49 30 18412-27300

e-mail: [Ralph.Pirow@bfr.bund.de](mailto:Ralph.Pirow@bfr.bund.de)

e-mail: [Andreas.Luch@bfr.bund.de](mailto:Andreas.Luch@bfr.bund.de)

**Table of Contents**

**Supplementary Tables**

Table S1 Dose-response data for the endpoint “foetal testosterone production ex vivo”

**Supplementary Figures**

Figure S1 Bayesian benchmark dose (BMD) modelling of the data of two experiments and of the combined data of both experiments

Figure S2 Histogram and density plot of the DnHexP concentrations in sunscreen

Figure S3 Proportion of primary and secondary metabolites in the total molar concentration of five metabolites of dibutyl phthalate (DBP)

Figure S4 Proportion of primary and secondary metabolites in the total molar concentration of three metabolites of diisobutyl phthalate (DiBP)

Figure S5 Proportion of primary and secondary metabolites in the total molar concentration of five metabolites of di(2-ethylhexyl) phthalate (DEHP)

Figure S6 Proportion of primary and secondary metabolites in the total molar concentration of three metabolites of DEHP

Figure S7 Proportion of primary and secondary metabolites in the total molar concentration of four metabolites of diisononyl phthalate (DiNP)

Figure S8 Proportion of primary and secondary metabolites in the total molar concentration of four metabolites of di(2-propylheptyl) phthalate (DPHP)

Figure S9 Proportion of primary and secondary metabolites in the total molar concentration of five metabolites of DEHP in dependency of the uptake pathway

**Evaluation of studies**

**Brief assessment of the use of 20% as an effect size for reduced testosterone production**

**Information on the urinary excretion fraction (*f*_UE_) of phthalate metabolites, the metabolite composition in urine, dermal absorption, and excretion via faeces**

**Supplemental Tables**

**Table S1** Dose-response data for the endpoint “foetal testosterone production ex vivo”. The data are from Fig. 1 of Saillenfait et al. (2013) and were kindly provided by Dr. Sophie Ndaw of the French National Research and Safety Institute for the Prevention of Occupational Accidents and Diseases (INRS).

| **Dose**  (mg/kg bw/day) | **Testosterone production**  **on gestational day 19**  (ng per testis per 3 h) | **Standard deviation** | **Number of litters** |
| --- | --- | --- | --- |
| 0 | 7.594 | 1.612 | 21 |
| 5 | 7.754 | 0.965 | 11 |
| 20 | 6.299 | 1.372 | 10 |
| 50 | 4.375 | 0.869 | 10 |
| 100 | 3.289 | 0.877 | 8 |
| 125 | 2.511 | 0.663 | 12 |
| 250 | 1.785 | 0.328 | 11 |
| 500 | 1.153 | 0.180 | 11 |
| 625 | 1.149 | 0.405 | 9 |

**Supplemental Figures**


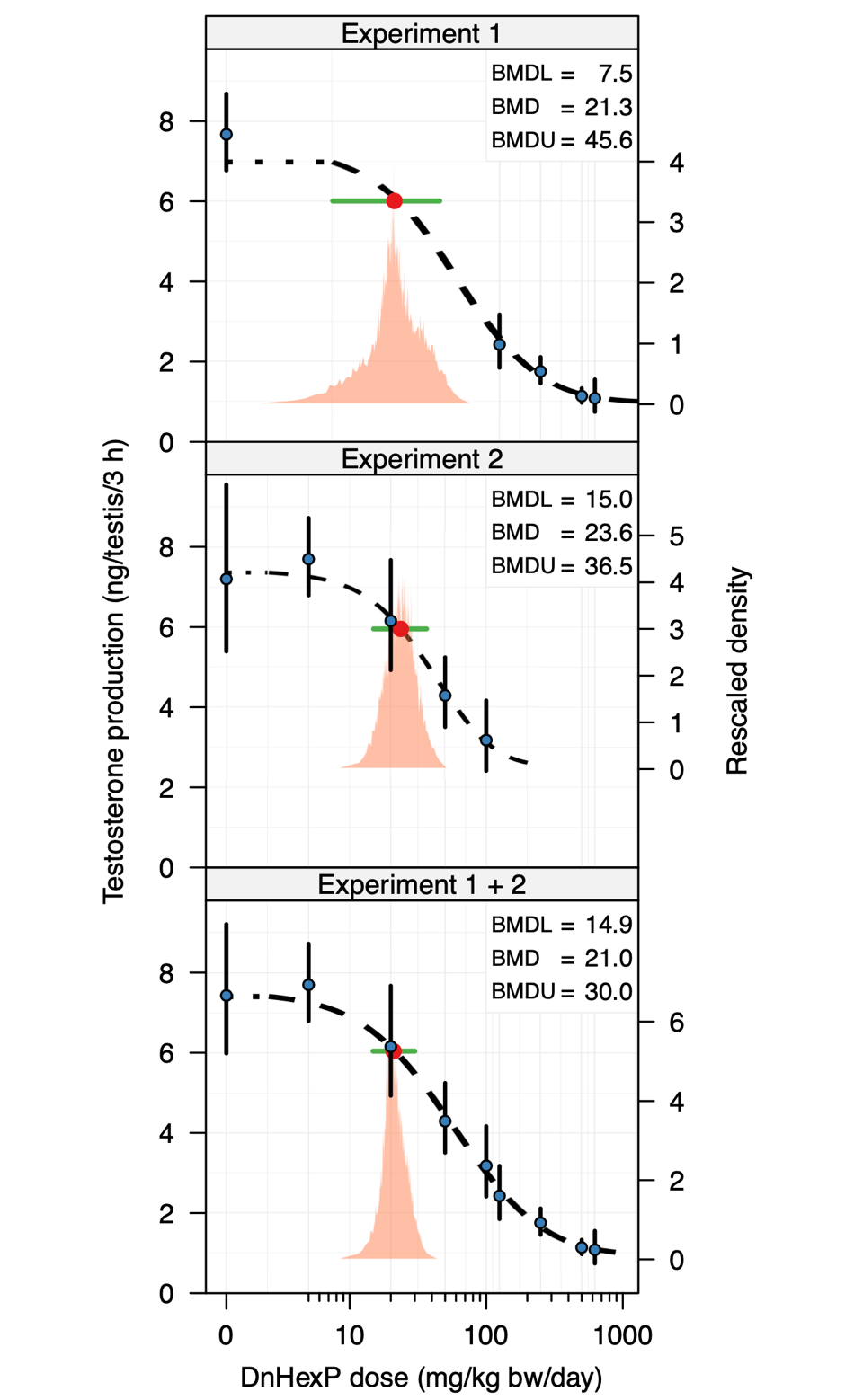


**Figure S1** Bayesian benchmark dose (BMD) modelling of the data of two experiments and of the combined data of both experiments. Foetal testosterone production ex vivo is plotted against the DnHexP dose administered to pregnant Sprague-Dawley rats in two separate experiments (Saillenfait et al. 2013). Blue-filled circle symbols and error bars show the geometric means and the (arithmetic) standard deviations. The dashed curves show the results of BMD modelling using Bayesian model averaging. Red dots and green bars indicate the model-averaged BMDs and the corresponding 95% credible intervals, respectively, for a benchmark response (BMR) of 20%. The orange areas represent the density distributions around the respective BMDs. Values for the BMD and for the lower and upper bounds (BMDL, BDMU) of the credible intervals are given.


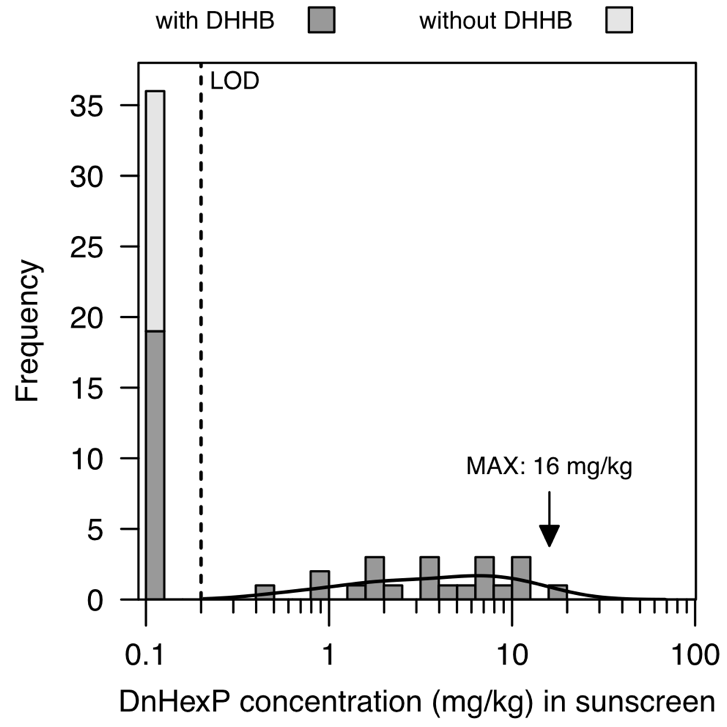


**Figure S2** Histogram and density plot of the DnHexP concentration in sunscreen. Data were obtained from the Chemical and Veterinary Research Office (CVUA) Karlsruhe. 57 sunscreen products with and without the UV filter hexyl 2-[4-(diethylamino)-2-hydroxybenzoyl]benzoate (EC no. 443-860-6, diethylamino hydroxybenzoyl hexyl benzoate, DHHB) were analysed. The maximum concentration (MAX) is indicated. The dashed line indicates the limit of detection (LOD) of 0.2 mg/kg. Values below the LOD are plotted at 0.5 × LOD.

**Supplemental Information: Evaluation of the studies**

Point of departure (PoD)

Studies were considered that were used in the dossier for the harmonised classification of DnHexP as toxic for reproduction according to Art. 37 of the CLP Regulation (ECHA 2011), as well as further studies published in the scientific literature. Therefore, common literature databases (Scopus, PubMed, Web of Science, Embase; Science Direct, Wiley) were searched for a combination of the following keywords:

- (("Dihexyl phthalate" OR "Di-n-hexyl phthalate" OR dihexylphthalate OR "201-559-5" OR "84-75-3") AND (toxic* OR harm* OR advers*OR poison* OR hazard*))
- (("Dihexyl phthalate" OR "Di-n-hexyl phthalate" OR dihexylphthalate OR "201-559-5" OR "84-75-3") AND ("reproduct* toxicity" OR developmental OR fecundity OR fertil* OR teratogen* OR testes OR testic* OR ovar* OR embryo* OR fetus* OR foetus* OR feti OR foetal OR fetal))
- (("Dihexyl phthalate" OR "Di-n-hexyl phthalate" OR dihexylphthalate OR "201-559-5" OR "84-75-3") AND (oestr* OR *estrus OR *estrous OR *fertili* OR steroid* OR acrosom* OR androgen* OR "anogenital distance" OR AGD OR areola OR "balanopreputial separation" OR breast OR cervi* OR "corpora lutea" OR "corpus luteum" OR "cryptorchidism" OR cumulus OR endocrin* OR endometr* OR epididym* OR estrogen* OR oestrogen* OR follic* OR FSH OR germ* OR gestation* OR gonad* OR granulosa OR hormon* OR hypospadia OR implantation* OR kisspeptin OR lactatio* OR Leydig OR luteal OR luteini* OR luteoly* OR mammar* OR menstrua* OR nipple* OR oocyt* OR oogenesis OR oogonia OR ovar* OR *ovulat* OR parturition OR pituitary OR polycystic OR preantral OR pregnan* OR primordial OR prolactin OR prostate OR pubert* OR reproduct* OR semen OR "seminiferous tubul*" OR Sertoli OR sex* OR "sexual maturation" OR sperm* OR testis OR testes OR testicul* OR testosteron* OR theca OR uterus OR *uterin* OR vagina*))

Only studies utilising in vivo exposure of mammals with at least three doses of DnHexP plus control group were considered for identification of potential PoDs. In total, 19 studies fulfilled these criteria. Based on the lowest effective doses, the following potential points of departure (PoDs) were identified for DnHexP:

Ye et al. (2020)

LO(A)EL (LOAEL = Lowest Observed Adverse Effect Level; the lowest dose at which an adverse effect was observed in the experiment. If the letter A is in brackets, this indicates that the observed effect is not considered adverse or that it is unclear whether the observed effect can be considered adverse): 10 mg/kg bw/day (Sprague-Dawley rats; exposure on postnatal days (PND) 35 – 56 to 0, 10, 100, and 1000 mg/kg bw/day DnHexP).

This LO(A)EL is based on a dose-dependent increase in the number of immunohistologically Cyp11a1-positive Leydig cells (Leydig cell hyperplasia) after exposure during puberty. A lack of blinding in the determination of cell counts is considered a deficiency.

Aydogan Ahbab and Barlas (2013), Aydogan Ahbab and Barlas (2015); Aydogan Ahbab et al. (2017); Aydogan Ahbab et al. (2014), Aydemir et al. (2023), Barlas et al. (2020) and Göktekin and Barlas (2017)

LOAEL: 20 mg/kg bw/day (Wistar rats; prenatal exposure on gestation days (GD) 6 – 19 with DnHexP at doses of 0, 20, 100, and 500 mg/kg bw/day).

This LOAEL is based, among other things, on decreased anogenital distance (AGD), decreased weight of the male reproductive organs and histopathological findings in the testes, prostate, epididymis and an increased number of abnormal sperm in the male offspring. Further findings concerned clinical serum parameters, haematology and thyroid histology. In many cases, a clear dose-response relationship was not discernible. The inclusion of litters in the statistics is unclear in some of the publications. Some of the publications (Aydemir et al. 2023; Aydogan Ahbab et al. 2017) refer to data from previous work by the same working group (Aydogan Ahbab and Barlas 2013; Aydogan Ahbab and Barlas 2015). Comparing the body weights of the offspring in the various publications, it is noticeable that these are identical for the dosed groups at the respective time points examined (PND 20, 32, 90). However, this is not the case for the control groups. This suggests that all publications are based on the same experiment, but that different control groups may have been used. The reliability of these publications is therefore considered to be limited.

Saillenfait et al. (2009)

NOAEL (No Observed Adverse Effect Level; in analogy to the LOAEL (see above), the highest dose at which no adverse effect was observed in the experiment): 50 mg/kg bw/day (prenatal exposure of Sprague-Dawley rats on GD 12 – 21 to 0, 50, 125, 250, and 500 mg/kg bw/day DnHexP).

This NOAEL is based on decreased AGD, increased nipple retention, reproductive tract malformations, and histopathological changes in reproductive organs in male offspring.

Substance identity and purity (> 98%) of DnHexP (test substance), as well as stability of the formulation (in olive oil according to the quality standard of the European Pharmacopoeia) were analytically verified.

DnHexP was administered to pregnant Sprague-Dawley rats (n = 9 – 13) by gavage (5 mL/kg bw) on GD 12 – 21 daily in the morning. The doses were adjusted to the body weights of the dams every 3 d. A variety of reproductive parameters were determined in male offspring including AGD (PND 1), retention of areolas/nipples (PND 12 – 14), time to preputial separation (PPS; from PND 40 onwards). Adult F1 males were sacrificed on PND 70 – 78 (all litters, three males per litter whenever possible) and on PND 111 – 120 (remaining males from each litter), and were examined for retention of nipples/areolas, and gross abnormalities of external and internal sexual organs. The weight of sexual organs was determined (PND 70 – 78), and histopathology was performed on testes and epididymides (PND 70 – 78 and 111 – 120). The litter was used as the experimental unit in the statistical analysis (ANOVA + Dunnett's test; or Kruskal-Wallis + Mann-Whitney; for AGD and PPS: ANCOVA + least significance test with body weight as covariate).

The study was considered robust with respect to the conduct and description of the study as well as the performed analyses. The statistical test power of nine to twelve litters is sufficient, and the litters were correctly considered as the relevant statistical unit. With regard to the parameters examined, the number of animals per group, and the duration of exposure, the experiments carried out do not comply with any OECD test guideline. However, this is without negative influence on the robustness of the study with regard to the research question (postnatal consequences of phthalate exposure on male reproductive development), because the exposure period of the pregnant rats covers the critical (phthalate-sensitive) phase during foetal development in male rats. Based on this study, a NOAEL of 50 mg/kg bw/day can be derived.

Furr et al. (2014)

NO(A)EL (No Observed Effect Level = dose at which test substance-related effects were not observed in the experiment, i.e., neither adverse nor non-adverse effects): 11 mg/kg bw/day (prenatal exposure of Sprague-Dawley rats on GD 12 – 21 to 0, 11, 100 or 300 mg DnHexP/kg bw/day).

This NO(A)EL is based on reduced testosterone production ex vivo. With three to four animals per dose group, the number of animals used is significantly lower than in the study by Saillenfait et al. (2013), in which eight animals per dose group were used. From this data, a BMDL_20_ in the range between 16 and 20 mg/kg bw/day was calculated, but it is statistically significantly less robust. It is also higher than the BMDL_20_ resulting from the study by Saillenfait et al. (2013) (14.7 mg/kg bw/day).

Saillenfait et al. (2013)

NO(A)EL: 5 mg/kg bw/day (prenatal exposure of Sprague-Dawley rats on GD 12 – 21 to 0, 5, 20, 50, 100, 125, 250, 500 or 625 mg/kg bw/day DnHexP).

This NO(A)EL is based on reduced testosterone production ex vivo. Substance identity and purity (> 98%) of DHP (test substance), as well as stability of the formulation (in olive oil according to the quality standard of the European Pharmacopoeia) were analytically verified.

The test substance was administered to pregnant Sprague-Dawley rats by gavage (5 mL/kg bw) on GD 12 – 19 daily in the morning. The doses were adjusted to the body weights of the dams every 3 d.

The following doses were administered in two experiments:

- DHP: 0, 125, 250, 500 or 625 mg kg bw/day and DEHP at 625 mg/kg bw/day

- DHP: 0, 5, 20, 50 or 100 mg kg bw/day and DEHP at 50 mg kg bw/day.

To determine testicular testosterone production, foetuses from eight to twelve litters (three foetuses per litter; two hormone measurements per foetus from the left and right testes) were used, and for gene expression analyses foetuses from eight litters (three foetuses per litter) were used.

In the high-dose experiment, the adrenal glands were also removed for gene expression analyses (eight litters, three foetuses per litter).

In addition, one testis per litter from three to six litters of the high-dose group was examined immunohistologically (Leydig cell and Sertoli cell markers).

As significant differences were not found between the control groups of both experiments (with regard to testicular testosterone production and gene expression), the respective control groups were combined to increase statistical power. The litter was used as the experimental unit in the statistical analysis (ANOVA + Dunnett's test).

Testicular ex vivo testosterone production was analysed after 3 h of incubation in culture medium using TCF-MS/MS (turbulent flow liquid chromatography coupled with tandem mass spectrometry). According to the authors, the method was established for the determination of testosterone in culture medium and was originally based on a method from Thermo Scientific for the determination of testosterone in blood plasma. The gene expression analysis was carried out using RT-PCR (SYBR-Green).

In conclusion, this is a robust study with respect to the conduct of the study and the analyses as well as their description. Although the number of exposed dams is unclear, the statistical test power of eight to twelve litters is sufficient, and the litters were correctly considered as the relevant statistical unit. Similar to the former study published in Saillenfait et al. (2009), the experiments carried out do not comply with any OECD test guideline but this is without negative influence on the robustness of the study with regard to the research question (phthalate effects on foetal testosterone production after prenatal exposure), because the exposure period of the pregnant rats covers the critical (phthalate-sensitive) phase during foetal development in male rats. Based on this study, a NO(A)EL of 5 mg/kg bw/day can be derived.

Although reduced foetal testicular testosterone production is not in itself an adverse effect, it is a well-established key event that ultimately leads to adverse effects in the male reproductive tract in rats (phthalate syndrome). Hence, the endpoint described in Saillenfait et al. (2013) represents a robust PoD and is taken forward for the risk assessment of DnHexP.

*Literature search for exposure via house dust*

A literature search was performed to obtain information on the possible origin of the source substance of MnHexP. For this purpose, common literature databases (Scopus, PubMed, Web of Science, Embase) were searched for a combination of the following keywords: „phthalate“; „hexyl“; „house dust“; „children“; „exposure“; “since 2016”. In a second round, the same databases were searched without the restriction "house dust" for the possible starting materials (including synonyms) for MnHexP. These were: „dihexyl phthalate“; „hexyl isotridecyl phthalate“; „hexyl isooctyl phthalate“; „hexyl octyl phthalate“ and "hexyl isodecyl phthalate".

**Supplemental Information: Brief assessment of the use of 20% as an effect size for reduced testosterone production**

In the short opinion of the HBM Commission of the German Federal Environment Agency (UBA 2024), a benchmark response (BMR) of 5% was used for BMD modelling without further scientific justification. A BMR of 5% may be considered overly conservative whereas a BMR of 20% is justified with respect to adversity and effect size theory.

A BMR of 20% resulted in a lower bound of the credible interval (BMDL) for the associated benchmark dose (BMD) of BMDL_20_ = 14.7 mg/kg bw/day. Reduced foetal testosterone production is an intermediate endpoint associated with adverse effects on male reproductive development. However, the corresponding effects in males such as decreased AGD, nipple retention, reproductive tract malformations, and histopathological changes in reproductive organs occurred at significantly higher doses (NOAEL 50 mg/kg bw/day) in the robust study by Saillenfait et al. (2009).

**Supplemental Information: Information on the urinary excretion fraction (*f*_UE_) of phthalate metabolites, the metabolite composition in urine, dermal absorption, and excretion via faeces**

The following sections compile the experimental evidence from peer-reviewed literature on relevant toxicokinetic aspects and parameters of different phthalates.

Fraction of urinary excretion and urine metabolite composition for different phthalates

This section summarises the available information on the fraction of urinary excretion (*f*_UE_) and urine metabolite composition for dibutyl phthalate (DBP), diisobutyl phthalate (DiBP), di(2-ethylhexyl) phthalate (DEHP), diisononyl phthalate (DiNP), and di(2-propylheptyl) phthalate (DPHP).

Oral administration of DBP to a person at a dose of ca. 60 µg/kg bw resulted in an urinary excretion of 92.2% of the dose within 24 h (Koch et al. 2012). The *f*_UE_ for the primary metabolite MBP was 0.84. Apart from MBP, four secondary metabolites were measured in urine. MBP accounted for more than 70% of the total molar concentration of all metabolites (Fig. S3).

Oral administration of DiBP to a person at a dose of ca. 60 µg/kg bw resulted in an urinary excretion of 90.3% of the dose within 24 h (Koch et al. 2012). The *f*_UE_ for the primary metabolite MiBP was 0.71. Apart from MiBP, two secondary metabolites were measured in urine. MiBP accounted for more than 50% of the total molar concentration of all metabolites (Fig. S4).

Oral administration of DEHP to a person at a dose of 641 µg/kg bw resulted in a urinary excretion of 47% of the dose within 44 h (Koch et al. 2004; Koch et al. 2005). The *f*_UE_ for the primary metabolite MEHP was 0.073. Apart from MEHP, four secondary metabolites were measured in urine. MEHP accounted for less than 20% of the total molar concentration of all metabolites (Fig. S5).

Oral administration of DEHP to a person at a dose of 645 µg/kg bw resulted in a urinary excretion of 31% of the dose within 46 h (Kessler et al. 2012). The *f*_UE_ for MEHP was 0.026. Apart from MEHP, two secondary metabolites were measured in urine. MEHP accounted for less than 15% of the total molar concentration of all metabolites (Fig. S6).

Oral administration of DiNP to a person at a dose of 1270 µg/kg bw resulted in an urinary excretion of 44% of the dose within 48 h (Koch and Angerer 2007). The *f*_UE_ was 0.022 for the primary metabolite MiNP. Apart from MiNP, three secondary metabolites were measured in urine. MiNP accounted for less than 10% of the total molar concentration of all metabolites (Fig. S7).

Oral administration of DPHP to six volunteers at a dose of 700 µg/kg bw resulted in a urinary excretion of 6% of the dose within 46 h (Klein et al. 2018). The *f*_UE_ was 0.0008 for the primary metabolite MPHP. Apart from MPHP, three secondary metabolites were measured in urine. MPHP accounted for less than 4% of the total molar concentration of all metabolites (Fig. S8).

To sum up and conclude, the recovery of excreted dose equivalents in urine decreases with increasing carbon number of the side chains, suggesting gastrointestinal absorption limitation and/or alternative, non-urinary (e. g., biliary) excretion pathways. The fraction of urinary excretion (*f*_UE_) of the primary metabolite is higher in phthalates with linear alkyl side chains compared to those with branched side chains of the same carbon number (cf. DBP *versus* DiBP). The *f*_UE_ decreases with increasing carbon number of the side chains (DiBP > DEHP > DiNP > DPHP). Likewise, the proportion of the primary metabolite in the total molar concentration of all metabolites in urine decreases with increasing carbon number of the side chains of branched-chain phthalates. Finally, the *f*_UE_ data suggest that the proportion of the primary metabolite is higher in linear-chain phthalates compared to branched-chain ones.

Urine metabolite composition in dependency of the exposure route

The phthalate metabolite composition in urine depends on the route of administration. Toxicokinetic data are available for the oral administration (Koch et al. 2004; Koch et al. 2005) and the combined dermal and inhalation exposure (Krais et al. 2018) of human volunteers to DEHP. The analysis of DEHP metabolite concentrations during the phase of maximum urinary excretion (1.5 – 11 h after oral administration, 4 – 7 h after dermal and inhalation exposure) revealed exposure route-dependent differences in the proportion of the primary metabolite MEHP in the total molar concentration of five metabolites of DEHP (Fig. S9). The proportion of MEHP was threefold higher following a combined dermal and inhalation exposure in comparison to oral exposure. This finding hints to differences in the first-pass metabolism for the oral and dermal/inhalation routes.

Skin permeation and metabolism of DEHP

The skin permeation and metabolism of DEHP was measured in vitro in excised human skin from patients undergoing abdominoplasty (Hopf et al. 2014). Emulsified DEHP in aqueous solution was applied to fresh dermatomed (800 µm) skin mounted on flow-through diffusion cells. The receptor fluid was analysed by HPLC-MS/MS. DEHP permeated viable human skin only as the primary metabolite MEHP. The application of emulsified MEHP in aqueous buffer resulted in a higher dermal permeation rate compared to the DnHexP; this experiment also showed that human skin is able to further oxidise MEHP to 5-oxo-MEHP.

Metabolism-dependent percutaneous absorption of DBP, BBzP, and DEHP

The percutaneous absorption of phthalates was measured in vitro in freshly excised, full-thickness skin from hairless rats with and without inhibition of skin esterases (Sugino et al. 2017). The phthalates were applied in 10% dimethyl sulphoxide in phosphate buffered saline to the skin mounted on diffusion cells. The receptor fluid was analysed by HPLC with UV/VIS detector. When DBP was applied to the skin, only the monoester MBP appeared through the skin, and skin permeability was higher than after the application of the MBP directly. The inhibition of skin esterases made the skin impermeable to MBP following dermal exposure to DBP. Similar phenomena were observed for benzyl butyl phthalate (BBzP). DEHP, which was not metabolised by esterases in the skin, was not transported across the skin. Overall, the data suggest that skin metabolism is essential for the percutaneous absorption of phthalates.

Urinary excretion of monoesters of DBP and DEP after repeated topical application

In a 2-week study, 26 volunteers received a daily topical application of basic cream (control week) and subsequently a cream containing 2% (w⁄w) of diethyl phthalate (DEP) and DBP each (treatment week) (Janjua et al. 2008). With a whole-body topical application at surface dose of 2 mg⁄cm^2^, the amount of cream per application ranged from 34 to 48 g (mean: 40 g) per participant. 24-h urine samples were collected, and the concentrations of unconjugated and total (unconjugated plus glucuronidated) mono-ethyl phthalate (MEP) and MBP were measured by LC-MS/MS. Of the applied DEP and DBP, 5.79% and 1.82%, respectively, could be recovered in urine as MEP and MBP. Of the total (unconjugated plus glucuronidated) amount of MEP and MBP, 78% and 8.0%, respectively, were excreted in the unconjugated form.

Urinary and faecal excretion of DEHP metabolites following the dermal exposure to a DEHP-plasticised PVC film

The in vivo percutaneous absorption of DEHP from a DEHP-plasticised PVC film was studied in rats (Deisinger et al. 1998). The PVC film was applied to the shaved backs of the animals for 24 h. Following the dermal exposure, the PVC film was removed and the exposed skin site was rewrapped to prevent the ingestion of any residual DEHP. Urine and faeces were collected for 7 days. For the experiments, ^14^C-radiolabelled DEHP was used. The amount of radioactivity transferred from the film was extremely small. Urinary excretion of radioactivity was 1.5-fold higher than the faecal excretion. The occurrence of radioactivity in the faeces suggests a partial excretion of DEHP metabolites via the bile and faeces.

Conclusions on the toxicokinetics and metabolism of phthalates

Competing reactions of phase-1 metabolism (ester cleavage, omega, omega-1, and β-oxidation of the side-chains), the route of administration, and the absorption through hepatic and lymphatic routes following oral exposure could be responsible for the fact that the metabolite composition in urine after oral administration differs from that after dermal (and inhalation) exposure. In the case of dermal exposure, the ester cleavage of the phthalate appears to be the dominant first step with formation of the monoester, which can then be further glucuronidated, but can also be transported in the unconjugated form via albumin in blood (similar to the free fatty acids), before becoming excreted.


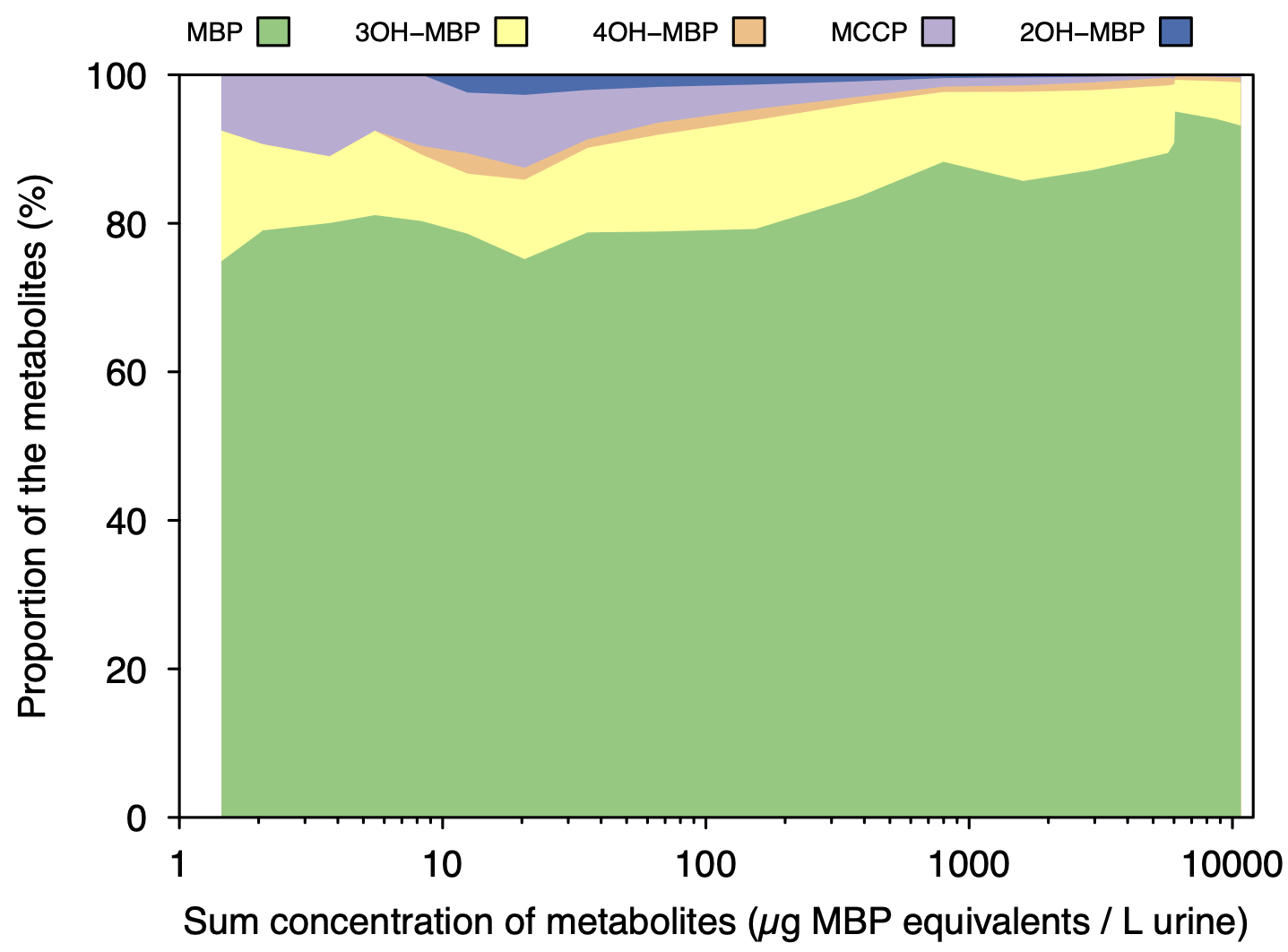


**Figure S3** Proportion of primary and secondary metabolites in the total molar concentration of five metabolites of dibutyl phthalate (DBP). Results were obtained from an analysis of the time-course of the metabolite concentrations in urine of a person who received an oral dose of ca. 60 µg/kg bw (Koch et al. 2012); data were excluded from the early absorption phase (≤ 1 h after administration). MCCP: 3-carboxy-mono-propyl phthalate.


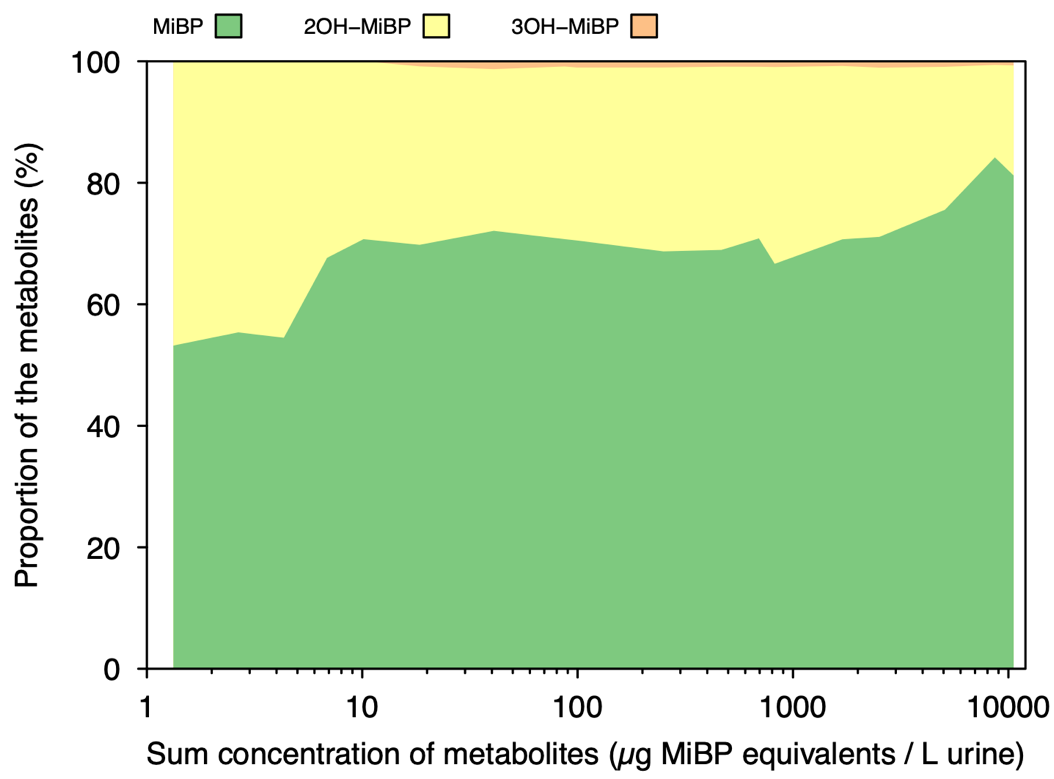


**Figure S4** Proportion of primary and secondary metabolites in the total molar concentration of three metabolites of diisobutyl phthalate (DiBP). Results were obtained from an analysis of the time-course of the metabolite concentrations in urine of a person orally dosed with ca. 60 µg/kg bw (Koch et al. 2012).


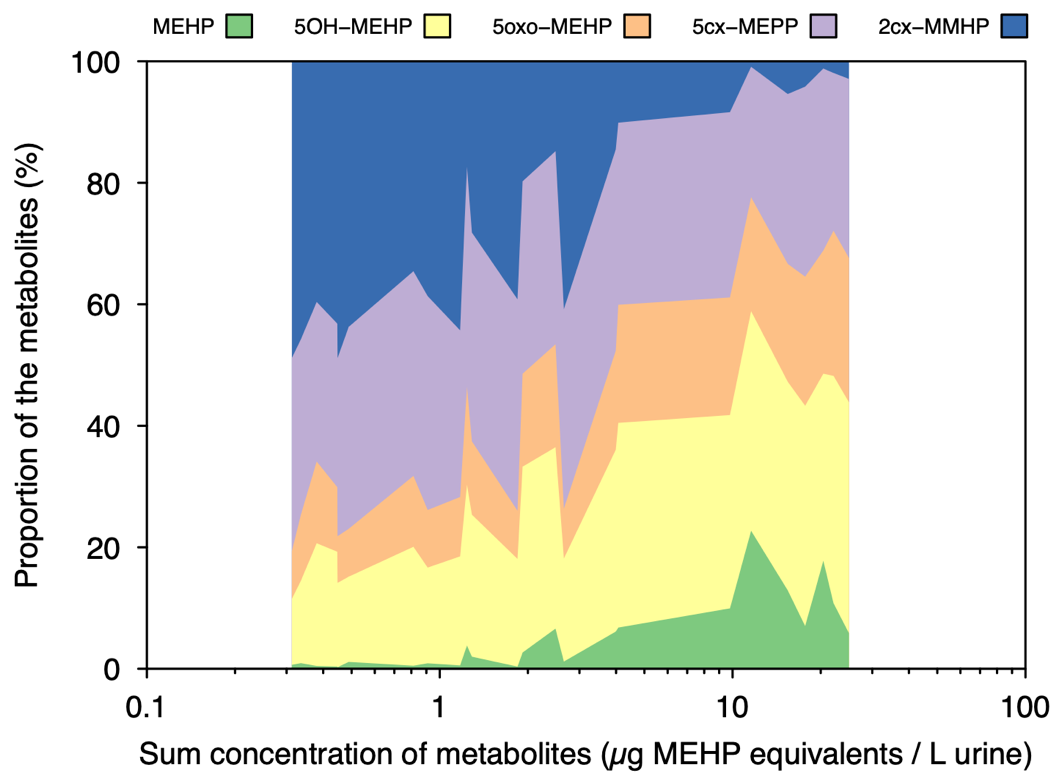


**Figure S5** Proportion of primary and secondary metabolites in the total molar concentration of five metabolites of di(2-ethylhexyl) phthalate (DEHP). Results were obtained from an analysis of the time-course of the metabolite concentrations in urine of a person who received an oral dose of 641 µg/kg bw (Koch et al. 2004; Koch et al. 2005). 5cx-MEPP: mono(2-ethyl-5-carboxypentyl) phthalate, 2cx-MMHP: mono[2-(carboxy­methyl), hexyl] phthalate.


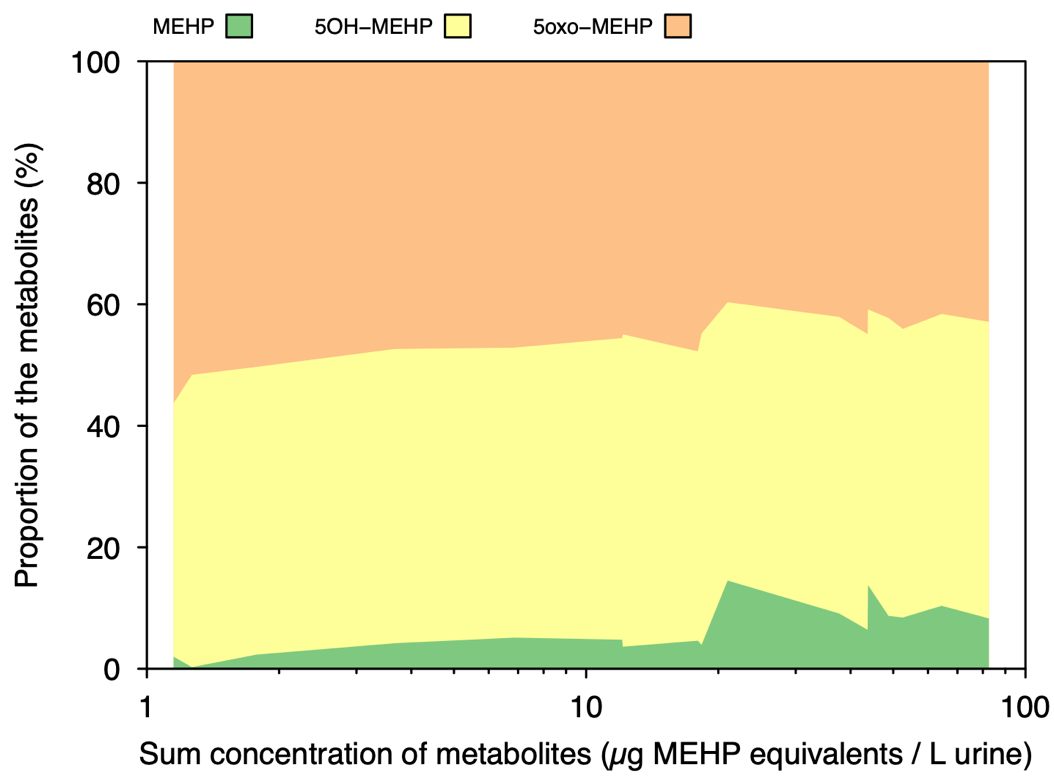


**Figure S6** Proportion of primary and secondary metabolites in the total molar concentration of three metabolites of DEHP. Results were obtained from an analysis of the time-course of the metabolite concentrations in urine of a person who received an oral dose of 645 µg/kg bw (Kessler et al. 2012).


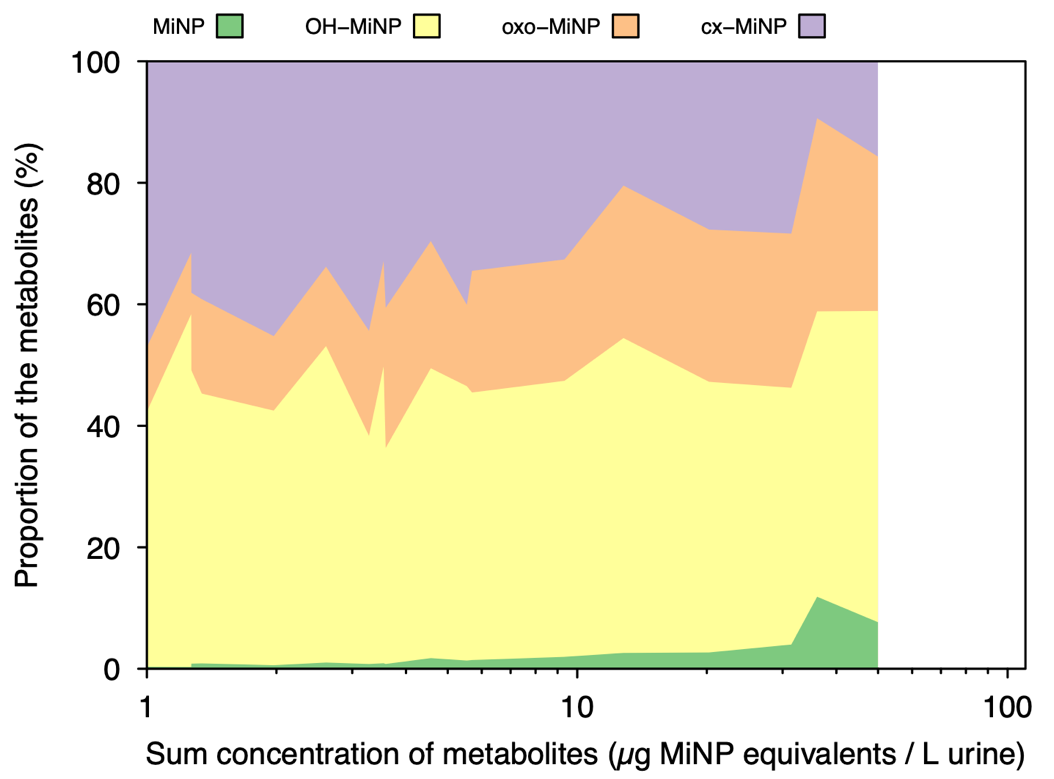


**Figure S7** Proportion of primary and secondary metabolites in the total molar concentration of four metabolites of diisononyl phthalate (DiNP). Results were obtained from an analysis of the time-course of the metabolite concentrations in urine of a person who received an oral dose of 1270 µg/kg bw (Koch and Angerer 2007).


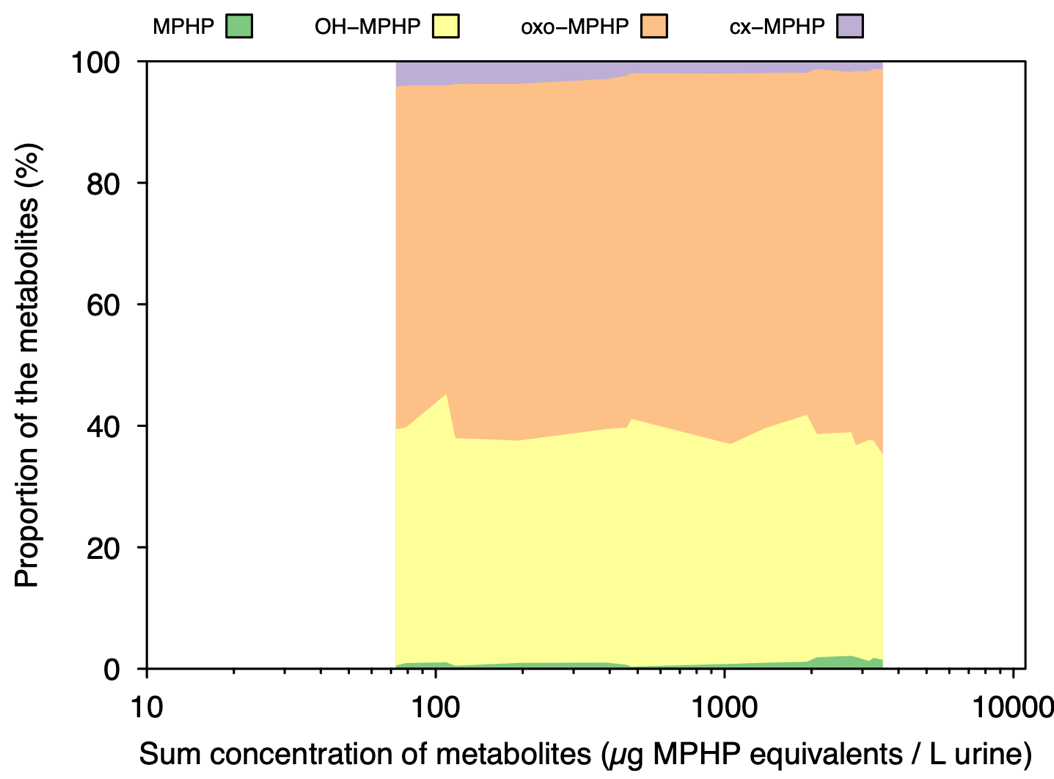


**Figure S8** Proportion of primary and secondary metabolites in the total molar concentration of four metabolites of di(2-propylheptyl) phthalate (DPHP). Results were obtained from an analysis of the time-course of the metabolite concentrations in the urine of six volunteers who received an oral dose of 700 µg/kg bw (Klein et al. 2018).


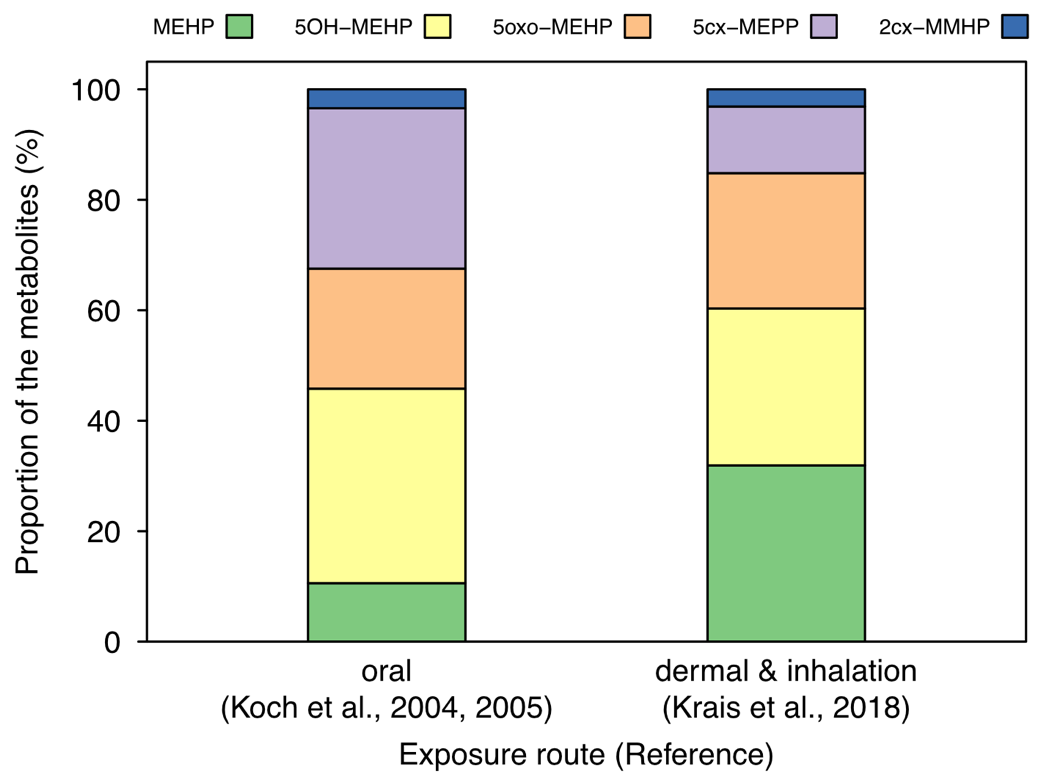


**Figure S9** Proportion of primary and secondary metabolites in the total molar concentration of five metabolites of DEHP in dependency of the uptake pathway. Results were obtained by an analysis of metabolite concentrations in the urine during the phase of maximum excretion (1.5 – 11 h after oral administration, 4 – 7 h after dermal and inhalation exposure). Data are taken from Koch et al. (2004), Koch et al. (2005) and Krais et al. (2018).

**References**

Aydemir D, Aydogan-Ahbab M, Barlas N, Ulusu NN (2023) Effects of the in-utero dicyclohexyl phthalate and di-n-hexyl phthalate administration on the oxidative stress-induced histopathological changes in the rat liver tissue correlated with serum biochemistry and hematological parameters. Front Endocrinol (Lausanne) 14:1128202 doi:10.3389/fendo.2023.1128202

Aydogan Ahbab M, Barlas N (2013) Developmental effects of prenatal di-n-hexyl phthalate and dicyclohexyl phthalate exposure on reproductive tract of male rats: Postnatal outcomes. Food Chem Toxicol 51:123-36 doi:10.1016/j.fct.2012.09.010

Aydogan Ahbab M, Barlas N (2015) Influence of in utero di-n-hexyl phthalate and dicyclohexyl phthalate on fetal testicular development in rats. Toxicol Lett 233(2):125-37 doi:10.1016/j.toxlet.2015.01.015

Aydogan Ahbab M, Guven C, Kockaya EA, Barlas N (2017) Comparative developmental toxicity evaluation of di- n-hexyl phthalate and dicyclohexyl phthalate in rats. Toxicol Ind Health 33(9):696-716 doi:10.1177/0748233717711868

Aydogan Ahbab M, Undeger U, Barlas N, Basaran N (2014) In utero exposure to dicyclohexyl and di-n-hexyl phthalate possess genotoxic effects on testicular cells of male rats after birth in the comet and TUNEL assays. Hum Exp Toxicol 33(3):230-9 doi:10.1177/0960327113494903

Barlas N, Goktekin E, Karabulut G (2020) Influence of in utero di-n-hexyl phthalate and di-cyclohexyl phthalate exposure on the endocrine glands and T3, T4, and TSH hormone levels of male and female rats: Postnatal outcomes. Toxicol Ind Health 36(6):399-416 doi:10.1177/0748233720931698

Deisinger PJ, Perry LG, Guest D (1998) In vivo percutaneous absorption of [14C]DEHP from [14C]DEHP-plasticized polyvinyl chloride film in male Fischer 344 rats. Food Chem Toxicol 36(6):521-7 doi:10.1016/s0278-6915(98)00015-5

ECHA (2011) Committee for Risk Assessment: Annex 1 - Background document to the Opinion proposing harmonised classification and labelling at Community level of Di-n-hexyl phthalate (DnHP). ECHA, ECHA/RAC/CLH-O-0000001541-83-03/A1. <https://echa.europa.eu/documents/10162/79437c82-d08e-bf60-38e8-d5acd42ca764>

Furr JR, Lambright CS, Wilson VS, Foster PM, Gray LE, Jr. (2014) A short-term in vivo screen using fetal testosterone production, a key event in the phthalate adverse outcome pathway, to predict disruption of sexual differentiation. Toxicol Sci 140(2):403-24 doi:10.1093/toxsci/kfu081

Göktekin E, Barlas N (2017) Biochemical and Histopathological Effects of in Utero Di-N-Hexyl Phthalate and Di-Cyclohexyl Phthalate Exposure on the Thyroid Axes and T3, T4, TSH Hormone Levels of Male and Female Rats: at Adulthood. Erciyes Med J 39(4):176-182 doi:10.5152/etd.2017.17069

Hopf NB, Berthet A, Vernez D, Langard E, Spring P, Gaudin R (2014) Skin permeation and metabolism of di(2-ethylhexyl) phthalate (DEHP). Toxicol Lett 224(1):47-53 doi:10.1016/j.toxlet.2013.10.004

Janjua NR, Frederiksen H, Skakkebaek NE, Wulf HC, Andersson AM (2008) Urinary excretion of phthalates and paraben after repeated whole-body topical application in humans. Int J Androl 31(2):118-30 doi:10.1111/j.1365-2605.2007.00841.x

Kessler W, Numtip W, Volkel W, et al. (2012) Kinetics of di(2-ethylhexyl) phthalate (DEHP) and mono(2-ethylhexyl) phthalate in blood and of DEHP metabolites in urine of male volunteers after single ingestion of ring-deuterated DEHP. Toxicol Appl Pharmacol 264(2):284-91 doi:10.1016/j.taap.2012.08.009

Klein D, Kessler W, Putz C, et al. (2018) Single ingestion of di-(2-propylheptyl) phthalate (DPHP) by male volunteers: DPHP in blood and its metabolites in blood and urine. Toxicol Lett 294:105-115 doi:10.1016/j.toxlet.2018.05.010

Koch HM, Angerer J (2007) Di-iso-nonylphthalate (DINP) metabolites in human urine after a single oral dose of deuterium-labelled DINP. Int J Hyg Environ Health 210(1):9-19 doi:10.1016/j.ijheh.2006.11.008

Koch HM, Bolt HM, Angerer J (2004) Di(2-ethylhexyl)phthalate (DEHP) metabolites in human urine and serum after a single oral dose of deuterium-labelled DEHP. Arch Toxicol 78(3):123-30 doi:10.1007/s00204-003-0522-3

Koch HM, Bolt HM, Preuss R, Angerer J (2005) New metabolites of di(2-ethylhexyl)phthalate (DEHP) in human urine and serum after single oral doses of deuterium-labelled DEHP. Arch Toxicol 79(7):367-76 doi:10.1007/s00204-004-0642-4

Koch HM, Christensen KL, Harth V, Lorber M, Bruning T (2012) Di-n-butyl phthalate (DnBP) and diisobutyl phthalate (DiBP) metabolism in a human volunteer after single oral doses. Arch Toxicol 86(12):1829-39 doi:10.1007/s00204-012-0908-1

Krais AM, Andersen C, Eriksson AC, et al. (2018) Excretion of Urinary Metabolites of the Phthalate Esters DEP and DEHP in 16 Volunteers after Inhalation and Dermal Exposure. Int J Environ Res Public Health 15(11) doi:10.3390/ijerph15112514

Saillenfait AM, Sabate JP, Gallissot F (2009) Effects of in utero exposure to di-n-hexyl phthalate on the reproductive development of the male rat. Reprod Toxicol 28(4):468-76 doi:10.1016/j.reprotox.2009.06.013

Saillenfait AM, Sabate JP, Robert A, et al. (2013) Dose-dependent alterations in gene expression and testosterone production in fetal rat testis after exposure to di-n-hexyl phthalate. J Appl Toxicol 33(9):1027-35 doi:10.1002/jat.2896

Sugino M, Hatanaka T, Todo H, et al. (2017) Safety evaluation of dermal exposure to phthalates: Metabolism-dependent percutaneous absorption. Toxicol Appl Pharmacol 328:10-17 doi:10.1016/j.taap.2017.05.009

UBA (2024) Assessment value set for plasticisers in urine - HBM Commission at the German Environment Agency publishes statement German Federal Environmental Agency, Dessau-Roßlau

Ye J, Zhang K, Yuan X, et al. (2020) Di-n-hexyl phthalate causes Leydig cell hyperplasia in rats during puberty. Toxicol Lett 332:213-221 doi:10.1016/j.toxlet.2020.07.018
